# Somatic morbidity in bipolar disorders

**DOI:** 10.1186/s40345-026-00427-9

**Published:** 2026-05-08

**Authors:** Christine Takami, Suvi Savinen, Arvid Sjölander, Zheng Chang, Isabell Brikell, Ralf Kuja-Halkola, Brian M. D’Onofrio, Soffia Gudbjornsdottir, Miguel Garcia-Argibay, Henrik Larsson, Erik Pettersson, Paul Lichtenstein, Mikael Landén

**Affiliations:** 1 Department of Medical Epidemiology and Biostatistics, Karolinska Institutet, Nobels väg 12A, Stockholm, 171 77 Sweden; 2 School of Educational Sciences and Psychology, University of Eastern Finland, Joensuu, Finland; 3 Department of Global Public Health and Primary Care, University of Bergen, Årstadveien 17, Bergen, 5009 Norway; 4 Department of Biomedicine, Aarhus University, Aarhus C, 8000 Denmark; 5 Department of Psychological and Brain Sciences, Indiana University, Bloomington, IN USA; 6 Department of Molecular and Clinical Medicine, Sahlgrenska Academy, University of Gothenburg, Goteborg, Sweden; 7 Developmental EPI (Evidence synthesis, Prediction, Implementation) lab, Centre for Innovation in Mental Health, Faculty of Environmental and Life Sciences, University of Southampton, Southampton, UK; 8 School of Medical Sciences, Faculty of Medicine and Health, Örebro University, Örebro, Sweden; 9 Institute of Neuroscience and Physiology, The Sahlgrenska Academy at Gothenburg University, Gothenburg, Sweden

**Keywords:** Bipolar disorder, Bipolar disorder type 1, Bipolar disorder type 2, Epidemiology, Somatic disease, Compulsory care

## Abstract

**Objective:**

This Swedish nationwide cohort study used large-scale data to investigate the associations between bipolar disorder and somatic disorders and whether these risks differ by subtype, sex, or exposure to compulsory care.

**Methods:**

61,071 individuals diagnosed with bipolar disorder in inpatient (from 1973) or outpatient care (from 2001) care were compared with the general population without bipolar disorder. The cohort included individuals born in 1932 or later, with follow-up from 1973 to 2020. Cox regression models estimated associations with a range of somatic conditions, including cardiovascular, endocrine, neurological, and infectious diseases. Subtype-specific analyses were conducted in individuals with type 1 (*n* = 8,352) or type 2 (*n* = 9,674), and in those with a history of compulsory care (*n* = 6,748).

**Results:**

Bipolar disorder was associated with significantly increased risks for most examined somatic conditions. The highest hazard ratios (HRs) were observed for sleep disorders (HR 3.79; 95% CI, 3.71–3.87) and dementias (HR 4.32; 95% CI, 3.82–4.79). Type 2 diabetes risk was elevated, while no association was found for type 1 diabetes. Most risks were comparable across bipolar subtypes, though certain conditions—such as migraine and fibromyalgia—were more strongly associated with type 2. Individuals with a history of compulsory psychiatric care showed elevated risks for several conditions.

**Conclusions:**

Regardless of sex or subtype, bipolar disorder is associated with substantially higher lifetime risks of a broad range of somatic conditions. Integrated psychiatric and somatic health care may help reduce morbidity and improve outcomes.

**Supplementary Information:**

The online version contains supplementary material available at 10.1186/s40345-026-00427-9.

## Introduction

Bipolar disorder is a chronic and severe mental disorder affecting 1–2% of the population (Merikangas et al. 2011). It is linked to an increased risk of obesity, type 2 diabetes, and other cardiometabolic and somatic conditions (Li et al. 2019, Silarova et al. 2015, Reilly-Harrington et al. 2018, Kessing et al. 2021, Vancampfort et al. 2015), all of which contribute to elevated rates of premature mortality (Sariaslan et al. 2022, Crump et al. 2013). While the clinical relevance of somatic-psychiatric multi-morbidity is increasingly acknowledged, important knowledge gaps remain. In particular, it is unclear how somatic risks vary between bipolar disorder subtypes or severity—distinctions that may have implications for tailoring prevention and treatment strategies.

Bipolar disorder is typically classified into two main subtypes—type 1 and 2—which differ in clinical manifestations and likely also in underlying etiology (Song et al. 2018, Karanti et al. 2020). The key distinguishing feature is the occurrence of manic episodes in type 1, characterized by severely elevated mood and in many cases psychotic features, not present in type 2.

While a recent study of 513 individuals aged ≥ 50 years found no significant differences in overall somatic burden between the subtypes (Beunders et al. 2023), comprehensive population-based data on the lifetime risks of specific somatic conditions by subtype remains scarce. In addition, it is unclear whether somatic risk profiles differ by sex or among individuals with more severe illness, such as those who have required compulsory care.

In this study, we used high-quality Swedish national registers, including the Swedish National Quality Register for Bipolar Disorder (BipoläR) and the Swedish National Diabetes Register, to investigate associations between bipolar disorder and 28 somatic conditions across cardiovascular, endocrine, neurological, and infectious disease domains. We assessed these associations overall and stratified by bipolar disorder subtype, sex, and history of compulsory psychiatric care.

## Methods

### Study population

We created a population-based cohort by linking data from multiple Swedish national registers using the unique personal identification number assigned to all residents. The cohort included all individuals born in 1932 or later, as identified through the Total Population Register (Ludvigsson et al. 2016). Individuals were excluded if they had emigrated before 1973, immigrated after 1973, or lacked maternal information in the Multi-Generation Register. A flow chart detailing the cohort assembly is provided in Supplementary Fig. 1.

### Exposure

Individuals with a bipolar disorder diagnosis were identified using the Swedish National Patient Register, which contains data on psychiatric inpatient care since 1973 and outpatient care since 2001. Bipolar disorder was defined as having at least two recorded diagnoses—based on ICD-8, ICD-9, or ICD-10 codes—within inpatient or outpatient settings. Specifically, we included the following codes: ICD-8: 296.00/10/20/30, 296.88/99; ICD-9: 296 A/B/C/D/E/W/X; and ICD-10: F30, F31. Two diagnoses of ICD-8 code 296.20 or ICD-9 code 296.B were not considered sufficient, as these codes may also capture unipolar depression. This case definition has been validated and shown to improve diagnostic accuracy (Sellgren et al. 2011). Individuals with only a single bipolar disorder diagnosis were excluded.

Bipolar disorder was treated as a lifetime exposure (Keramatian et al. 2022); individuals with a confirmed diagnosis were considered exposed from birth onward. To avoid misclassification, individuals with any diagnosis of schizophrenia (ICD-8: 295.00–295.60.00.60, 295.80, 295.99; ICD-9: 295 A-295G, 295 W, 295X; ICD-10: F20) were excluded from the comparison group without bipolar disorder.

The Swedish national quality assurance register for bipolar disorders (BipoläR) has collected clinician-reported data from psychiatric outpatient services since 2004 (Pålsson et al. 2022). Using this register, we identified individuals diagnosed with bipolar disorder type 1 or type 2 within a subset of the overall bipolar disorder population, as subtype information is not available in the National Patient Register. Individuals with documented diagnosis of both subtypes (*n* = 2,162) were classified as having bipolar disorder type 1. The population comparison group and the mode of analysis for the subtype analyses were the same as for the main analysis. However, individuals with bipolar disorder not represented in the quality register were excluded.

To approximate illness severity, individuals with any history of compulsory care (*n* = 6,748) were analyzed as a separate group.

### Outcomes and covariates

Information on somatic disorders was retrieved from the National Patient Register, which has near complete coverage of diagnoses made in specialized care since 1987 and outpatient specialized care since 2001 (Ludvigsson et al. 2011). The selection of somatic conditions was informed by previous literature documenting associations with bipolar disorder.

We used parental educational level as a proxy for early-life socioeconomic status, based on the lifetime highest level of educational attained by either parent, as recorded in the Longitudinal Integration Database for Health Insurance and Market Studies (Ludvigsson et al. 2019). The most recent year of data available was 2019.

To distinguish between type 1 and 2 diabetes, we supplemented data from the National Patient Register with information from the Swedish National Diabetes Register (NDR), established in 1996. This distinction was not available in the National Patient Register throughout the entire follow-up period. Use of NDR was particularly important given that metabolic risk factors are primarily associated with type 2 diabetes. Additionally, the NDR captures diagnoses made in primary care, where most cases of type 2 diabetes are managed.

### Statistical analyses

We estimated absolute risks and risk differences from birth among individuals born between 1970 and 1975 (*n* = 6,738), comparing those with and without bipolar disorder. This birth cohort was selected to ensure comparable follow-up times across individuals.

Cox proportional hazards models, with age in years as the underlying time-scale, were then used to estimate hazard ratios (HRs) for each outcome in the full cohort, including analyses stratified by sex. Follow-up began on January 1, 1973, or at birth for those born later, and ended at the time of death, emigration, or December 31, 2020.

All analyses were adjusted for year of birth and highest parental education level (categorized as compulsory school, upper secondary school, or university/higher education). To account for potential clustering of siblings, standard errors were adjusted for clustering by the mother’s identifier. Main analyses were also adjusted for region of birth.

We similarly used Cox regression models to examine associations within the subset of individuals identified in the BipoläR quality register, with analyses further stratified by bipolar disorder subtype and sex.

We conducted two sensitivity analyses to assess the robustness of our findings. First, we treated bipolar disorder as a time-varying exposure, with exposure starting at the date of the first recorded diagnosis (while still requiring at least two diagnoses). This approach enables comparison with previous studies and examined whether associations differed when follow-up began at diagnosis rather than birth. Second, to address potential Berkson bias—where individuals with bipolar disorder may receive somatic diagnoses more frequently in specialized care compared to the general population—we restricted analyses to main diagnoses only. This reduces the likelihood of capturing secondary diagnoses that might otherwise go undocumented in primary care, from which we lack data. All models were implemented using SAS version 9.4 (SAS Institute, Cary, NC, USA) and STATA version 17.0.

## Results

### Demographics

Among the 7,973,091 individuals in the cohort, 61,071 (0.8%) met the criteria for bipolar disorder (Table 1). Women comprised 63% of those with bipolar disorder. The proportion of individuals with at least one parent holding a university-level education was slightly lower in the bipolar disorder group (34%) compared to those without the disorder (40%). Parental educational levels were comparable across bipolar disorder subtypes.

**Table 1:** Sociodemographic characteristics of the study population

	Bipolar disorder all *n* (%)	Not having bipolar disorder *n* (%)	Women with bipolar disorder *n* (%)	Women not having bipolar disorder *n* (%)	Men with bipolar disorder *n* (%)	Men not having bipolar disorder *n* (%)
**Sex**						
Women	38 157 (63%)	3 834 165 (49%)	N/A	N/A	N/A	N/A
**Highest educational level of parent**						
Compulsory school^1^	13 787 (23%)	1 697 895 (21%)	16 137 (21%)	825 922 (21%)	5 691 (21%)	871 973 (21%)
Upper secondary school	26 463 (43%)	3 077 370 (39%)	16,999 (45%)	1 491 385 (39%)	9 464 (41%)	1 585 985 (39%)
University or higher	20 821 (34%)	3 136 755 (40%)	13 062 (34%)	1 516 858 (40%)	7 759 (34%)	1 619 897 (40%)
Total	61 071	7 912 020	38 157	3 834 165	22 914	4 077 855

	**Having bipolar I disorder n (%)**	**Not having bipolar I disorder (%)**	**Having bipolar II disorder n (%)**	**Not having bipolar II disorder n (%)**	**Bipolar disorder with compulsory care n (%)**	**Bipolar disorder outpatient care ** **n (%)**
**Sex**						
Women	4 903 (59%)	3 834 061 (49%)	5 151 (69%)	3 833 907 (49%)	298 (55%)	27 432 (64%)
**Highest educational level of parent**						
Compulsory school^1^	2 012 (24%)	1 697 865 (21%)	1 332 (18%)	1 697 848 (21%)	129 (24%)	8178 (19%)
Upper secondary school	3 278 (39%)	3 0 77 300 (39%)	3 278 (44%)	3 077 206 (39%)	223 (41%)	19 068 (44%)
University or higher	3 062 (37%)	3 136 660 (40%)	2 862 (38%)	3 136 577 (40%)	195 (35%)	15 755 (37%)
Total	8 352	7 911 825	7 512	7 911 631	547	43 001

^1^ 9 years of schooling or less; N/A = Not applicable

### Absolute risks and risk differences

Table 2 presents the absolute risks (counts and percentages) and corresponding risk differences for all studied outcomes among individuals born between 1970 and 1975, comparing those with and without bipolar disorder.

**Table 2:** Absolute risk and risk difference in individuals born 1970–1975 in those with a lifetime diagnosis of bipolar disorder (*N* = 6,738; women *N* = 4,257) and in the population without bipolar disorder (*N* = 590,465; women *N* = 284,658)

Outcomes	With bipolar disorder *n* (%)	No bipolar disorder *n* (%)	Risk difference (%) (95% CI)	Men with bipolar disorder *n* (%)	Men with no bipolar disorder *n* (%)	Risk difference (%) (95% CI)	Women with bipolar disorder *n* (%)	Women with no bipolar disorder *n* (%)	Risk difference (%) (95% CI)
**Cardiovascular and cerebrovascular conditions**									
Cardiovascular disease	657 (9.75)	34,634 (5.87)	3.89 (3.17–4.60)	260 (10.48)	19,281 (6.30)	4.17 (2.97–5.38)	397 (9.33)	15,353 (5.39)	3.93 (3.05–4.81)
Hyperlipidemia	150 (2.23)	7,673 (1.30)	0.93 (0.57–1.28)	72 (2.90)	5,438 (1.78)	1.12 (0.46–1.79)	78 (1.83)	2,235 (0.79)	1.05 (0.64–1.45)
Hypertensive diseases	584 (8.67)	25,145 (4.26)	4.41 (3.73–5.08)	247 (9.96)	14,849 (4.86)	5.10 (3.92–6.28)	337 (7.92)	10,296 (3.62)	4.30 (3.49–5.11)
Arteriosclerosis	62 (0.92)	3,744 (0.63)	0.29 (0.06–0.51)	24 (0.97)	1,955 (0.64)	0.33 (−0.06–0.71)	38 (0.89)	1,789 (0.63)	0.26 (−0.02–0.55)
Ischemic heart diseases	66 (0.98)	4,136 (0.70)	0.28 (0.04–0.52)	34 (1.37)	2,951 (0.96)	0.41 (−0.05–0.86)	32 (0.75)	1,185 (0.42)	0.34 (0.07–0.60)
Arrhythmias	194 (2.88)	11,572 (1.96)	0.92 (0.52–1.32)	86 (3.47)	7,157 (2.34)	1.13 (0.40–1.85)	108 (2.54)	4,415 (1.55)	0.99 (0.51–1.46)
Heart failure	58 (0.86)	3,274 (0.55)	0.31 (0.08–0.53)	31 (1.25)	2,172 (0.71)	0.54 (0.10–0.98)	27 (0.63)	1,102 (0.39)	0.25 (0.01–0.49)
Thromboembolic disease	254 (3.77)	11,285 (1.91)	1.86 (1.40–2.31)	84 (3.39)	5,213 (1.70)	1.68 (0.97–2.39)	170 (3.99)	6,072 (2.13)	1.86 (1.27–2.45)
Cerebrovascular disease	167 (2.48)	7,216 (1.22)	1.26 (0.88–1.63)	68 (2.74)	3,868 (1.26)	1.48 (0.83–2.12)	99 (2.33)	3,348 (1.18)	1.15 (0.69–1.60)
**Infectious**,** inflammatory**,** or autoimmune conditions**									
Bacterial infection	3,161 (46.91)	162,029 (27.44)	19.47 (18.28–20.67)	773 (31.16)	61,513 (20.11)	11.04 (9.21–12.87)	2,388 (56.10)	100,516 (35.31)	20.78 (19.28–22.29)
Viral infection	945 (14.02)	42,639 (7.22)	6.80 (5.97–7.64)	324 (13.06)	20,728 (6.78)	6.28 (4.95–7.61)	621 (14.59)	21,911 (7.70)	6.89 (5.83–7.96)
COVID-19	50 (0.74)	2,633 (0.45)	0.30 (0.09–0.50)	12 (0.48)	1,271 (0.42)	0.07 (−0.021–0.34)	38 (0.89)	1,362 (0.48)	0.41 (0.13–0.70)
Autoimmune disease	786 (11.67)	45,019 (7.62)	4.04 (3.27–4.81)	219 (8.83)	18,794 (6.15)	2.68 (1.56–3.80)	567 (13.32)	26,225 (9.21)	4.11 (3.08–5.13)
Asthma	632 (9.38)	26,562 (4.50)	4.88 (4.18–5.58)	169 (6.81)	11,573 (3.78)	3.03 (0.20–0.40)	463 (10.88)	14,989 (5.27)	5.61 (4.67–6.55)
Type 1 diabetes (NPR)	119 (1.63)	6,814 (1.15)	0.61 (0.30–0.93)	53 (2.14)	3,939 (1.29)	0.85 (0.28–1.42)	66 (1.55)	2,875 (1.01)	0.54 (0.17–0.91)
Type 1 diabetes (NDR)	75 (1.11)	5,447 (0.92)	0.19 (−0.06–0.44)	35 (1.41)	3,135 (1.03)	0.39 (−0.08–0.85)	40 (0.94)	2,312 (0.81)	0.13 (−0.016–0.42)
Type 2 diabetes (NDR)	440 (6.53)	14,970 (2.54)	3.99 (3.40–4.59)	196 (7.90)	9,583 (3.13)	4.77 (3.70–5.83)	244 (5.73)	5,387 (1.89)	3.84 (3.14–4.54)
**Neurological conditions**									
Alzheimer disease	9 (0.13)	129 (0.02)	0.11 (0.02–0.20)	N/A	N/A	N/A	N/A	N/A	N/A
Other dementias	N/A	N/A	N/A	N/A	N/A	N/A	N/A	N/A	N/A
Epilepsy	272 (4.04)	9,145 (1.55)	2.49 (2.02–2.96)	104 (4.19)	4,771 (1.56)	2.36 (1.84–3.42)	168 (3.95)	4,374 (1.54)	2.41 (1.82–3.00)
Migraine	463 (6.87)	18,272 (3.09)	3.78 (3.17–4.38)	70 (2.82)	4,842 (1.58)	1.24 (0.59–1.89)	393 (9.23)	13,430 (4.72)	4.51 (3.64–5.39)
Sleep disorders	966 (14.34)	18,900 (3.20)	11.14 (10.30–11.97)	407 (16.40)	13,239 (4.33)	12.08 (10.62–13.53)	559 (13.13)	5,661 (1.99)	11.14 (10.13–12.16)
**Autoimmune**,** endocrinological**,** and other conditions**									
Polycystic ovary syndrome	N/A	N/A	N/A	N/A	N/A	N/A	102 (2.40)	3,604 (1.27)	1.13 (0.67–1.59)
Obesity	884 (13.12)	24,828 (4.20)	8.91 (8.11–9.72)	159 (6.41)	7,049 (2.31)	4.10 (3.14–5.07)	725 (17.03)	17,779 (6.25)	10.79 (9.65–11.92)
Restless legs syndrome	39 (0.58)	863 (0.15)	0.43 (0.25–0.61)	9 (0.36)	368 (0.12)	0.24 (0.01–0.48)	30 (0.70)	495 (0.17)	0.53 (0.28–0.78)
Irritable bowel syndrome	334 (4.96)	9,224 (1.56)	3.39 (2.88–3.91)	63 (2.54)	2,738 (0.90)	1.64 (1.02–2.26)	271 (6.37)	6,486 (2.28)	4.09 (3.35–4.82)
Fibromyalgia	237 (3.52)	4,454 (0.75)	2.76 (2.32 − 3.20)	11 (0.44)	229 (0.07)	0.37 (0.11–0.63)	226 (5.31)	4,225 (1.48)	3.82 (3.15–4.50)
Chronic fatigue syndrome	32 (0.47)	658 (0.11)	0.36 (0.20–0.53)	5 (0.20)	164 (0.05)	0.15 (−0.03–0.32)	27 (0.63)	494 (0.17)	0.46 (0.22–0.70)

N/A = not applicable or not displayed due to less than 5 affected cases; NPR = The National Patient Register; NDR = The National Diabetes Register

The largest absolute risk differences were observed for bacterial infections (approximately 20%), sleep disorder (11%), and obesity (9%). With respect to sex differences, women with bipolar disorder had higher rates of obesity, asthma, and infections compared with men. As the maximum age in this subset was 50 years, the number of dementia diagnoses was low.

### Relative risks

#### All with bipolar disorder

**Fig. 1:**
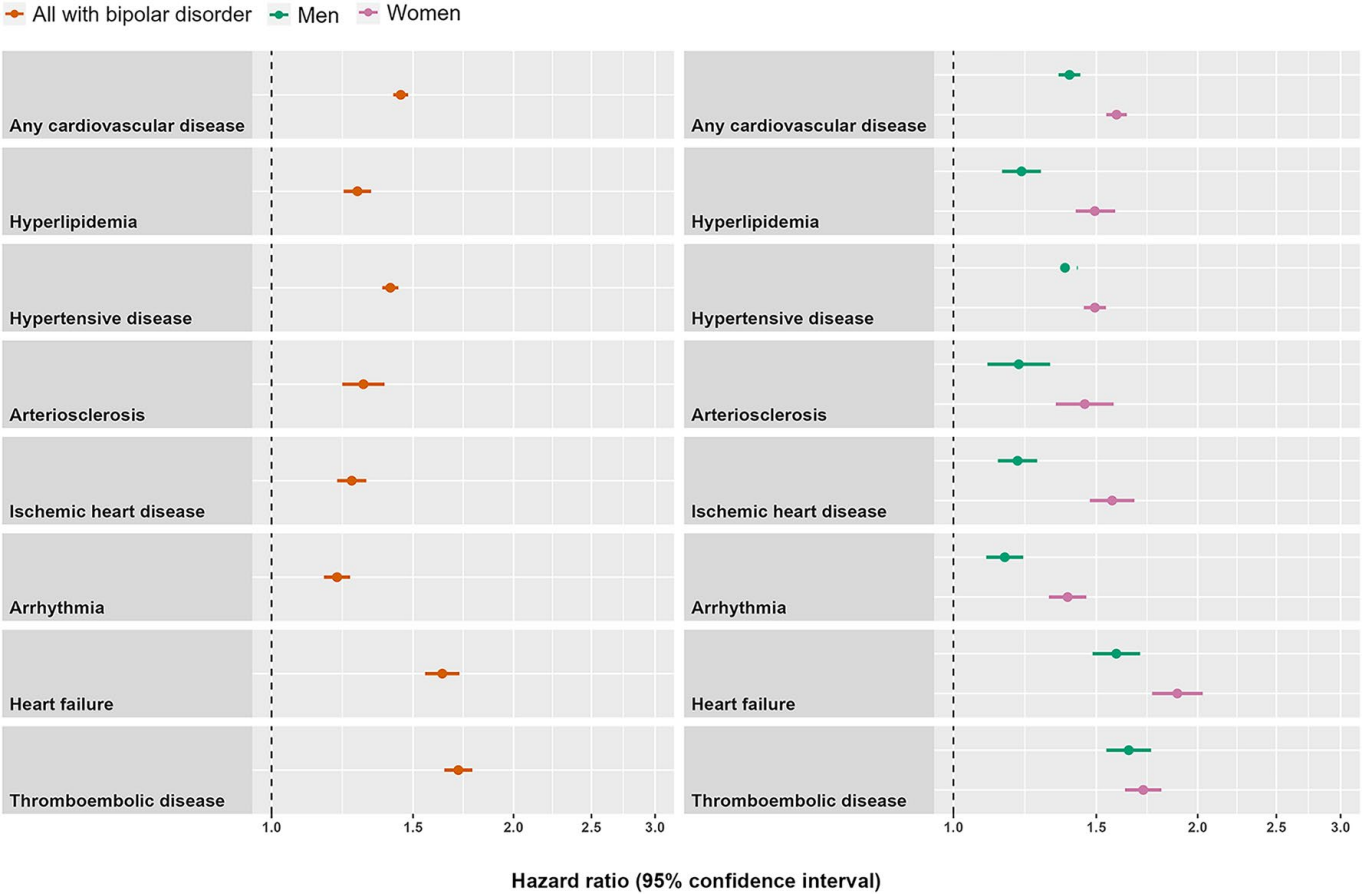
Cardiovascular conditions in individuals with bipolar disorder (*N* = 61,071) compared with the background population (*N* = 7,912,020), in men with bipolar disorder (*N* = 22,914) and without bipolar disorder (*N* = 4,077,855) and women with (*N* = 38,157) and without bipolar disorder (*N* = 3,834,165)

**Fig. 2:**
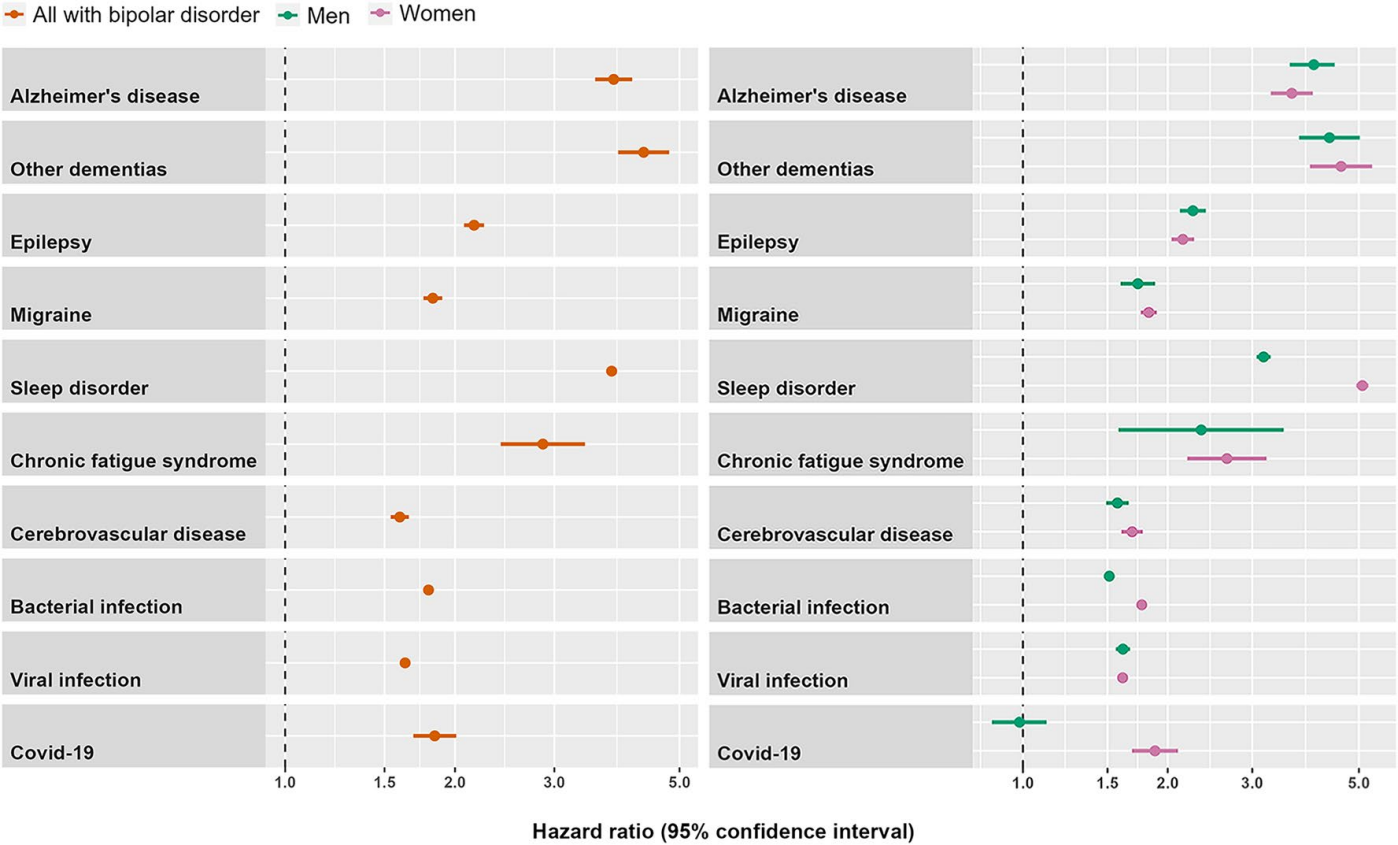
Autoimmune, endocrinological and other conditions in individuals with bipolar disorder (*N*=61,071) compared with the background population (*N*=7,912,020), in men with bipolar disorder (*N*=22,914) and without bipolar disorder (*N*=4,077,855) and women with (*N*=38,157) and without bipolar disorder (*N*=3,834,165)

**Fig. 3:**
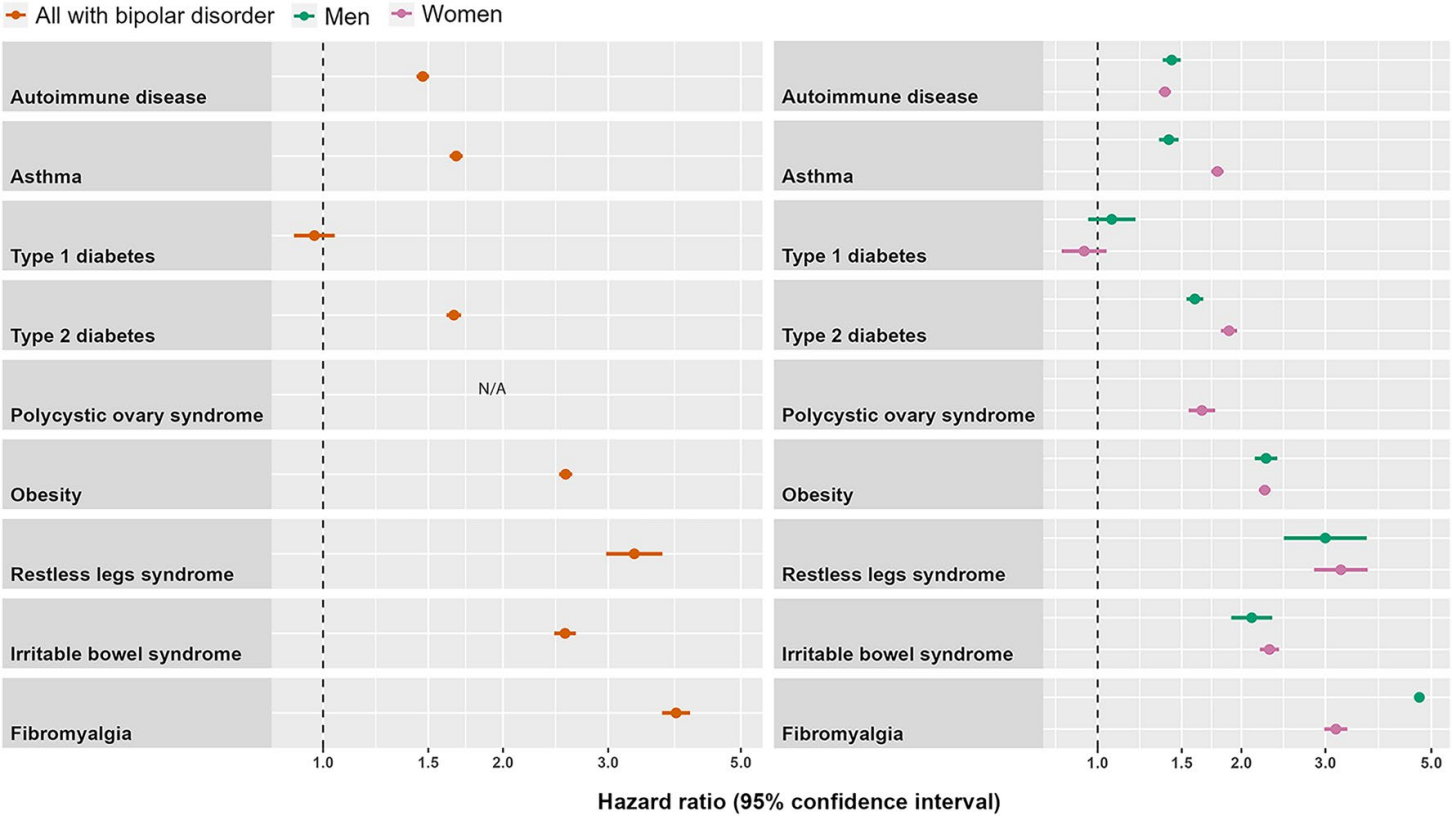
Neurological conditions and infectious diseases in individuals with bipolar disorder (*N*=61,071) compared with the background population (*N*=7,912,020), in men with bipolar disorder (*N*=22,914) and without bipolar disorder (*N*=4,077,855) and women with (*N*=38,157) and without bipolar disorder (*N*=3,834,165)

Individuals with bipolar disorder had higher relative risks for almost all examined outcomes compared with the general population (Figs. 1, 2 and 3). Cardiovascular conditions were notably elevated, with HRs ranging from 1.21 for arrhythmias to 2.06 for peripheral artery disease (Fig. 1 and Supplementary Table 1). In sex stratified analyses, women had a slightly higher relative risk increases than men for hyperlipidemia, peripheral artery disease, ischemic heart disease, and heart failure. However, the absolute risks for these conditions remained lower in women than in men with bipolar disorder (Table 2).

The association between bipolar disorder and type 1 diabetes was statistically significant when using data from the National Patient Register. However, this association was not statistically significant—or only borderline significant—when using the more diagnostically precise and more comprehensive National Diabetes Register (Fig. 2 and Supplementary Table 1). In contrast, the risk for type 2 diabetes was consistently increased in both men and women with bipolar disorder.

Elevated risks were also observed for bacterial and viral infectious diseases, including COVID-19 (Fig. 3 and Supplementary Table 1).

Individuals with bipolar disorder had 3–4-fold increased risk of Alzheimer’s disease and other dementias (Fig. 3 and Supplementary Table 1). Elevated risks were also observed for epilepsy, migraine, sleep disorder, and chronic fatigue syndrome, with comparable estimates for men and women.

#### Bipolar disorder subtypes

Analyses by bipolar disorder subtypes revealed that both type 1 and type 2 were associated with the majority of somatic outcomes (Table 3). Notable exceptions included arteriosclerosis, which was significantly associated only with bipolar disorder type 2 in both sexes. Similarly, the risk of ischemic heart disease was elevated only in type 2 overall, although it was also elevated among women with type 1 in sex-stratified analyses. In contrast, heart failure was significantly associated only in women with bipolar disorder type 1.

Type 1 diabetes showed no clear association with either bipolar disorder subtype, with results being borderline or non-significant. In contrast, type 2 diabetes was significantly associated with both subtypes irrespective of sex.

Bipolar disorder type 2 was associated with higher risk estimates for migraine and fibromyalgia than type 1. The risks for Alzheimer’s disease and other dementias were similar across subtypes. Sleep disorders were also markedly elevated in both bipolar disorder subtypes, with HRs approaching 5 among women.

**Table 3:** Associations between bipolar disorder and somatic outcomes, presented by bipolar disorder subtype and sex

Outcomes	All with bipolar I disorder HR (95% CI)	Men with bipolar I disorder HR (95% CI)	Women with bipolar I disorder HR (95% CI)	All with bipolar disorder type 2 HR (95% CI)	Men with bipolar disorder type 2 HR (95% CI)	Women with bipolar disorder type 2 HR (95% CI)
**Cardiovascular conditions**						
Any cardiovascular disease	1.19 (1.13–1.26)	1.17 (1.08–1.26)	1.29 (1.20–1.40)	1.21 (1.12–1.29)	1.28 (1.15–1.42)	1.24 (1.13–1.36)
Hyperlipidemia	1.14 (1.03–1.26)	1.15 (1.01–1.32)	1.24 (1.07–1.44)	1.41 (1.25–1.59)	1.46 (1.24–1.73)	1.58 (1.33–1.87)
Hypertensive diseases	1.36 (1.28–1.43)	1.32 (1.22–1.43)	1.44 (1.33–1.55)	1.30 (1.21–1.41)	1.25 (1.12–1.41)	1.42 (1.29–1.57)
Arteriosclerosis	0.96 (0.81–1.14)	0.90 (0.71–1.55)	1.07 (0.85–1.36)	1.40 (1.16–1.69)	1.39 (1.05–1.85)	1.45 (1.14–1.86)
Ischemic heart diseases	0.99 (0.88–1.10)	0.92 (0.79–1.06)	1.27 (1.07–1.50)	1.22 (1.06–1.40)	1.31 (1.09–1.58)	1.39 (1.12–1.72)
Arrhythmia	1.01 (0.92–1.12)	0.99 (0.86–1.13)	1.15 (0.99–1.32)	0.92 (0.81–1.06)	0.91 (0.75–1.11)	1.05 (0.88–1.26)
Heart failure	1.24 (1.08–1.42)	1.16 (0.96–1.40)	1.51 (1.24–1.83)	1.09 (0.90–1.33)	1.13 (0.86–1.48)	1.24 (0.94–1.63)
Thromboembolic disease	1.63 (1.47–1.80)	1.69 (1.45–1.96)	1.56 (1.37–1.79)	1.50 (1.32–1.71)	1.55 (1.26–1.92)	1.42 (1.21–1.67)
Cerebrovascular diseases	1.39 (1.26–1.53)	1.50 (1.32–1.71)	1.34 (1.16–1.53)	1.42 (1.25–1.61)	1.51 (1.25–1.82)	1.43 (1.20–1.69)
**Infectious**,** inflammatory**,** or autoimmune conditions**						
Bacterial infection	1.66 (1.61–1.72)	1.42 (1.34–1.51)	1.69 (1.63–1.76)	1.75 (1.70–1.81)	1.36 (1.26–1.46)	1.67 (1.61–1.74)
Viral infection	1.41 (1.33–1.50)	1.24 (1.12–1.37)	1.51 (1.40–1.62)	1.37 (1.29–1.45)	1.36 (1.21–1.52)	1.34 (1.25–1.44)
COVID-19	1.64 (1.30–2.05)	1.73 (1.23–2.43)	1.55 (1.15–2.09)	2.41 (1.93–3.02)	1.65 (1.04–2.63)	2.68 (2.08–3.46)
Autoimmune disease	1.54 (1.45–1.64)	1.57 (1.41–1.74)	1.45 (1.34–1.56)	1.49 (1.39–1.59)	1.21 (1.05–1.40)	1.44 (1.34–1.56)
Asthma	1.52 (1.41–1.64)	1.29 (1.13–1.47)	1.62 (1.48–1.77)	1.63 (1.52–1.75)	1.42 (1.23–1.64)	1.69 (1.55–1.83)
Type 1 diabetes NPR	1.18 (1.02–1.37)	1.14 (0.92–1.41)	1.30 (1.06–1.58)	1.02 (0.85–1.22)	1.16 (0.89–1.52)	0.99 (0.78–1.27)
Type 1 diabetes NDR	0.94 (0.75–1.18)	0.94 (0.67–1.31)	0.99 (0.73–1.34)	0.98 (0.78–1.23)	1.41 (1.02–1.94)	0.81 (0.59–1.17)
Type 2 diabetes NDR	1.99 (1.86–2.12)	1.88 (1.71–2.06)	2.29 (2.08–2.51)	1.81 (1.65–1.98)	1.70 (1.49–1.94)	2.14 (1.90–2.41)
**Neurological conditions**						
Alzheimer disease	3.57 (2.95–4.32)	3.99 (3.00–5.29)	3.24 (2.51–4.18)	3.17 (2.36–4.26)	3.36 (2.08–5.42)	2.99 (2.05–4.36)
Other dementias	3.22 (2.41–4.31)	3.25 (2.16–4.89)	3.43 (2.26–5.21)	2.23 (1.34–3.70)	2.61 (1.30–5.43)	2.08 (0.99–4.37)
Epilepsy	1.60 (1.41–1.81)	1.54 (1.27–1.86)	1.70 (1.44–2.00)	1.61 (1.40–1.84)	1.22 (0.94–1.58)	1.87 (1.60–2.18)
Migraine	1.62 (1.46–1.79)	1.35 (1.06–1.71)	1.50 (1.34–1.69)	2.32 (2.12–2.53)	1.82 (1.43–2.33)	1.97 (1.79–2.18)
Sleep disorders	3.53 (3.34–3.74)	2.97 (2.74–3.24)	4.82 (4.46–5.21)	3.75 (3.53–3.99)	3.44 (3.12–3.80)	4.82 (4.45–5.22)
**Other somatic conditions**						
Polycystic ovary syndrome	N/A	N/A	1.75 (1.45–2.13)	N/A	N/A	1.71 (1.45–2.00)
Obesity	2.43 (2.27–2.61)	2.01 (1.74–2.33)	2.30 (2.13–2.49)	2.76 (2.58–2.95)	2.36 (2.00–2.78)	2.28 (2.12–2.45)
Restless legs syndrome	2.73 (2.03–3.67)	2.71 (1.63–4.50)	2.58 (1.79–3.72)	4.14 (3.10–5.54)	5.42 (3.37–8.73)	3.29 (2.29–4.75)
Irritable bowel syndrome	2.18 (1.94–2.46)	1.89 (1.45–2.46)	2.03 (1.78–2.32)	2.75 (2.46–3.07)	2.50 (1.90–3.30)	2.29 (2.03–2.59)
Fibromyalgia	2.45 (2.07–2.90)	0.90 (0.22–3.60)	2.13 (1.79–2.53)	4.11 (3.55–4.76)	4.75 (2.26–9.98)	3.08 (2.65–3.58)
Chronic fatigue syndrome	1.81 (1.03–3.19)	1.22 (0.31–4.89)	1.80 (0.97–3.35)	2.52 (1.52–4.18)	1.76 (0.44–7.05)	2.25 (1.30–3.88)

N/A=not applicable or too few cases (<5)

Results presented as hazard ratios (HR) with 95% confidence intervals adjusted for parental highest educational level and year of birth. HRs represent estimates comparing bipolar disorder type 1 (N=8,352; women N=4,903) and bipolar disorder type 2 (N=9,674; women N=6,498) to individuals without any bipolar disorder diagnoses

#### Compulsory care

Individuals with bipolar disorder who had experienced psychiatric compulsory care had markedly higher risks for several outcomes. Notably, HRs were over seven for Alzheimer’s disease (HR 7.22; 95% CI, 6.19–8.43) and nearly seven for other dementias (HR 6.93; 95% CI, 5.50–8.72) (Supplementary Table 1). Elevated risks were also observed for thromboembolic disease (HR 2.34; 95% CI, 2.12–2.58) and COVID-19 (HR 2.39; 95% CI, 1.92–2.97).

#### Sensitivity analyses

The sensitivity analysis treating bipolar disorder as a time-varying exposure yielded results largely consistent with the main analysis. Exceptions included lower risk estimates for fibromyalgia and restless legs syndrome, and a tendency towards higher risk estimates for epilepsy (Supplementary Table 3).

In the sensitivity analysis restricted to main diagnoses only, HRs remained statistically significant but were generally lower for cardiovascular outcomes, including hyperlipidemia, hypertension, and peripheral artery disease (Supplementary Table 3). Similar attenuation was observed for Alzheimer’s disease, sleep disorder, and restless legs syndrome.

To assess the proportional hazards assumption, we estimated time-varying hazard ratios over age for bipolar disorder, adjusted for covariates as in the main model (Supplemental Figs. 2–4). Schoenfeld residuals are presented in Supplemental Figs. 5 to 7.

## Discussion

We conducted a population-based cohort study to investigate risks of a broad spectrum of somatic conditions in individuals with bipolar disorder, with analyses stratified by sex, disorder subtype, and illness severity. The results revealed significantly increased risks for almost all investigated outcomes in both men and women with bipolar disorder. Given the lifetime exposure definition of bipolar disorder, the reported associations should be interpreted as reflecting lifetime co-occurrence patterns rather than risks following clinical onset. Accordingly, these findings do not in themselves permit causal interpretation.

Importantly, this is the first large-scale study to examine somatic outcomes separately for bipolar disorder types 1 and 2. While most outcomes showed similar risk patterns across subtypes, several notable differences emerged. Individuals with bipolar disorder type 2 had higher risks for certain cardiovascular conditions, including hyperlipidemia and arteriosclerosis, as well as for migraine and fibromyalgia, compared with those with type 1. When differences between the subtypes were observed, risk estimates were generally higher for type 2, with two exceptions: autoimmune diseases were more common in men with type 1, and heart failure was associated only with women with type 1. These findings are somewhat unexpected, given that bipolar disorder type 1 is typically regarded as the more severe subtype based on manic symptoms, hospitalization rates, and functional impairment. However, the observed somatic burden in type 2 suggests that the conventional markers of psychiatric severity may not fully capture the dimensions relevant to physical health. Type 2 is often characterized by more frequent or prolonged depressive episodes, which are associated with increased systemic inflammation, metabolic dysregulation, and chronic pain—factors that may underlie the elevated risks of cardiometabolic and pain-related conditions in this group. Thus, somatic comorbidity may align more closely with the chronicity and nature of mood symptoms than with traditional measures of psychiatric severity. Furthermore, subtype differences may result from differences in health care management, treatment, or comorbidity patterns (Karanti et al. 2020).

The overall risk of cardiovascular disease was elevated in individuals with bipolar disorder, with the highest relative risks observed for peripheral artery disease, cerebrovascular diseases, and thromboembolic disease. These findings align with those of a Danish register-based study that included individuals with bipolar disorder and their unaffected siblings (Kessing et al. 2021). A potential biological mechanism involves low-grade inflammation, which has been implicated in both bipolar disorder and vascular pathology. Elevated serum concentrations of interleukin-6—a proinflammatory cytokine—have been observed during hypomanic and manic episodes (Munkholm et al. 2015, Velazquez-Salinas et al. 2019), and are known to contribute to vascular dysfunction and atherosclerosis (Rosenblat and McIntyre 2017, Ramji and Davies 2015). Consistent with a heightened vascular risk profile, a study of adolescents with early-onset psychosis or bipolar disorder found structural changes in the left common carotid artery, suggestive of preclinical arteriosclerosis, compared with controls (Bohman et al. 2020).

Pro-inflammatory cytokines also influence lipid metabolism and glucose metabolism. We speculate that such pathophysiological mechanisms could contribute to the increased risks of hyperlipidemia, obesity, and type 2 diabetes that we observed in bipolar disorder. Our findings align with a systematic review and meta-analysis reporting that approximately one third of individuals with bipolar disorder meet criteria for metabolic syndrome, defined as central obesity plus at least two of elevated blood glucose, blood pressure, or abnormal lipid levels (Vancampfort et al. 2013). In prior work, we found that such cardiometabolic risk indicators in individuals with bipolar disorder remained elevated—or increased further—over a 6–7 year follow-up compared with healthy controls (Najar et al. 2022, Najar et al. 2023). The twofold HR for obesity in the current study observed in the present study aligns with recent findings that individuals with bipolar disorder not only have higher body mass index (BMI), but also experience greater BMI increases over time, than the general population (Najar et al. 2023). While lifestyle factors may contribute to this elevated risk, weight gain due to psychotropic medications could play a role (Najar et al. 2017).

The link between bipolar disorder and immune system aberrations is further supported by the elevated risks of asthma, diabetes type 1, other autoimmune diseases, and both bacterial and viral infections. These findings align with prior research linking bipolar disorder to immune dysregulation and increased vulnerability to inflammatory conditions (Kuś et al. 2021, Cremaschi et al. 2017, Cyranka et al. 2023, Wu et al. 2016, Munkholm et al. 2013). The likely reason why type 1 diabetes was only statistically significant when using National Patient Register data is that the register did not differentiate between diabetes subtypes prior to 1997.

Consistent with previous studies (Sucksdorff et al. 2015, Romo-Nava et al. 2021), we found elevated lifetime risks of epilepsy and migraine among individuals with bipolar disorder. However, we are aware of the possibility that these conditions may precede rather than follow the onset of bipolar disorder. This may be the reason for the difference in risk estimates seen in the time-varying sensitivity analysis (Supplementary Table 2). By contrast, Alzheimer’s disease and other forms of dementia typically manifest later in life, after a bipolar disorder diagnosis. We found a threefold increase in relative risk for both Alzheimer’s disease and other dementias, slightly exceeding estimates reported in a Danish cohort study (Kessing et al. 2021). Notably, the association was even stronger among individuals who had experienced compulsory psychiatric care, likely reflecting a subgroup with more severe illness. Several mechanisms may contribute to the elevated risk of dementia in this population, including comorbid alcohol use disorder (Rehm et al. 2019), potential neurotoxic effects of recurrent manic episodes (Elefante et al. 2023), and lifestyle-related factors such as physical inactivity.

We observed an elevated risk of polycystic ovary syndrome (PCOS) across all investigated groups with bipolar disorder. This association may be influenced by the mood stabilizer valproate, which can disrupt hormonal regulation and lead to features of PCOS. However, PCOS may also increase the risk for bipolar disorder, as suggested by a previous study reporting increased risks of several psychiatric conditions—including bipolar disorder—following a diagnosis of PCOS (Cesta et al. 2016).

Fibromyalgia and chronic fatigue syndrome were approximately three times more prevalent among individuals with bipolar disorder, which aligns with previous findings (Kudlow et al. 2015). Notably, however, the absolute risks for fibromyalgia and chronic fatigue syndrome were below 1%. Restless legs syndrome was also three times more prevalent. This association could be influenced by side effects of medications used in bipolar disorder, such as lithium (Michopoulos et al. 2014).

### Strengths and limitations

This study has several key strengths. The use of comprehensive Swedish national registers ensured high data quality, broad population coverage, and minimal loss to follow-up. The large sample size and long observation period increase generalizability and enabled robust estimations of associations across a wide range of somatic conditions. Additionally, inclusion of data from the National Quality Register for Bipolar Disorders (BipoläR) allowed for a rare opportunity to examine somatic morbidity by bipolar disorder subtype, which is typically not feasible in register-based studies.

There are also limitations that warrant consideration. First, as this is an observational study, causal inferences cannot be made, and residual confounding may influence the results. The results should be interpreted as associations with the first recorded diagnosis of each outcome, rather than as capturing the full burden or temporal sequence of multimorbidity. Individuals may contribute to multiple outcomes across analyses, and repeated events or trajectories of somatic conditions were not assessed. Second, individuals with bipolar disorder in Sweden typically receive annual blood testing in specialized psychiatric care, which may increase the likelihood of detecting conditions such as hyperlipidemia, potentially leading to overestimation of relative risks. In the same vein, all patients with bipolar disorder in this study had engaged with specialized psychiatric care, where they might have received collateral diagnoses of somatic conditions, potentially leading to overestimation of risks. Reassuringly a sensitivity analysis restricted to main diagnoses showed largely similar estimates (see Supplementary Table 3), though some risk inflation due to Berkson bias cannot be excluded. Additionally, the absence of primary care data may limit the generalizability of our findings, particularly for common conditions—such as hypertension, hyperlipidemia, migraine, obesity, mild infections and dementia—that are often diagnosed and managed outside of specialized care. Supporting this concern, the observed association with type 1 diabetes was present using the National Patient Register but not in the more comprehensive National Diabetes Register. This is particularly relevant for conditions like dementia, which are frequently managed outside of specialist settings. Furthermore, outpatient data were only available from 2001 onward, meaning earlier follow-up captures only inpatient diagnoses and more severe cases. Furthermore, we cannot rule out the possibility of selection bias in the BipoläR-based analyses as inclusion in the register may be influenced by healthcare utilization and clinical characteristics.

Last, generalizability to other countries may be limited by differences in health care systems.

## Conclusion

Bipolar disorder is associated with increased lifetime risks across a wide range of somatic conditions. While most risks were similar across subtypes, bipolar disorder type 2 was particularly associated with higher risks for migraine and fibromyalgia. Elevated risks in individuals with a history of compulsory care suggest greater somatic burden in more severe illness. These findings underscore the need for a multi-morbidity approach and stronger integration between psychiatric and somatic healthcare to improve outcomes for individuals with bipolar disorder.

## Supplementary Information

Supplementary Material 1

## Data Availability

Data for this study are not freely available upon demand due to regulations imposed by the Swedish national register holders to protect the personal integrity of the persons whose data were used.
