# Correction: Somatic morbidity in bipolar disorders

**DOI:** 10.1186/s40345-026-00432-y

**Published:** 2026-06-30

**Authors:** Christine Takami, Suvi Savinen, Arvid Sjölander, Zheng Chang, Isabell Brikell, Ralf Kuja-Halkola, Brian M. D’Onofrio, Soffia Gudbjornsdottir, Miguel Garcia-Argibay, Henrik Larsson, Erik Pettersson, Paul Lichtenstein, Mikael Landén

**Affiliations:** 1 Department of Medical Epidemiology and Biostatistics, Karolinska Institutet, Nobels väg 12A, Stockholm, 171 77 Sweden; 2 School of Educational Sciences and Psychology, University of Eastern Finland, Joensuu, Finland; 3 Department of Global Public Health and Primary Care, University of Bergen, Årstadveien 17, Bergen, 5009 Norway; 4 Department of Biomedicine, Aarhus University, Aarhus C, 8000 Denmark; 5 Department of Psychological and Brain Sciences, Indiana University, Bloomington, IN USA; 6 Department of Molecular and Clinical Medicine, Sahlgrenska Academy, University of Gothenburg, Goteborg, Sweden; 7 Developmental EPI (Evidence synthesis, Prediction, Implementation) lab, Centre for Innovation in Mental Health, Faculty of Environmental and Life Sciences, University of Southampton, Southampton, UK; 8 School of Medical Sciences, Faculty of Medicine and Health, Örebro University, Örebro, Sweden; 9 Institute of Neuroscience and Physiology, The Sahlgrenska Academy at Gothenburg University, Gothenburg, Sweden

**Correction: International Journal of Bipolar Disorders (2026) 14:19**

10.1186/s40345-026-00427-9

In this article (Takami et al. 2026), Fig. 2 appeared incorrectly and have now been corrected in the original publication. For completeness and transparency, both correct and incorrect versions are displayed below.

The original article has been corrected.

Incorrect Figure 2

**Fig. 2:**
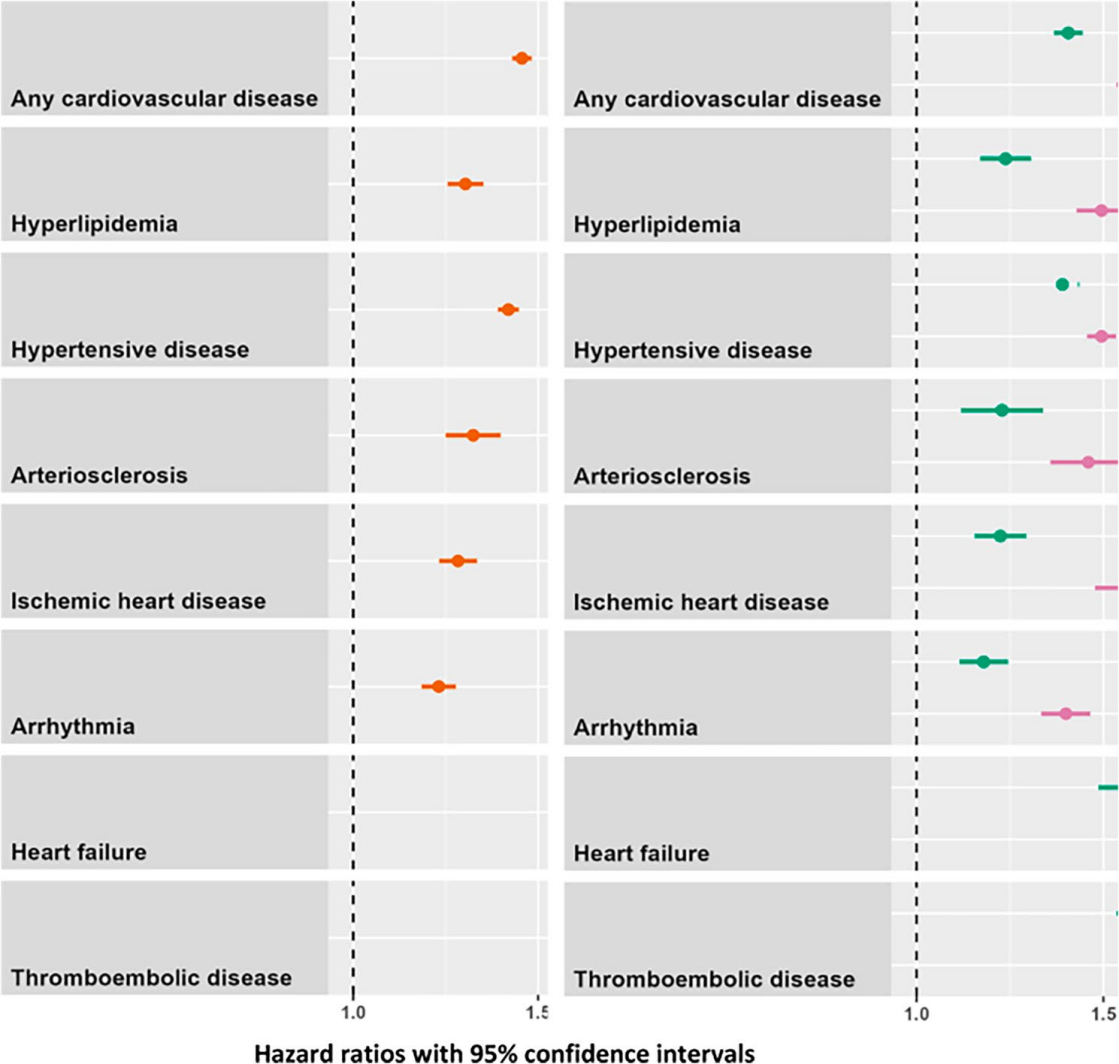
Autoimmune, endocrinological and other conditions in individuals with bipolar disorder (N=61,071) compared with the background population (N=7,912,020), in men with bipolar disorder (N=22,914) and without bipolar disorder (N=4,077,855) and women with (N=38,157) and without bipolar disorder (N=3,834,165)

Correct Figure 2

**Fig. 2:**
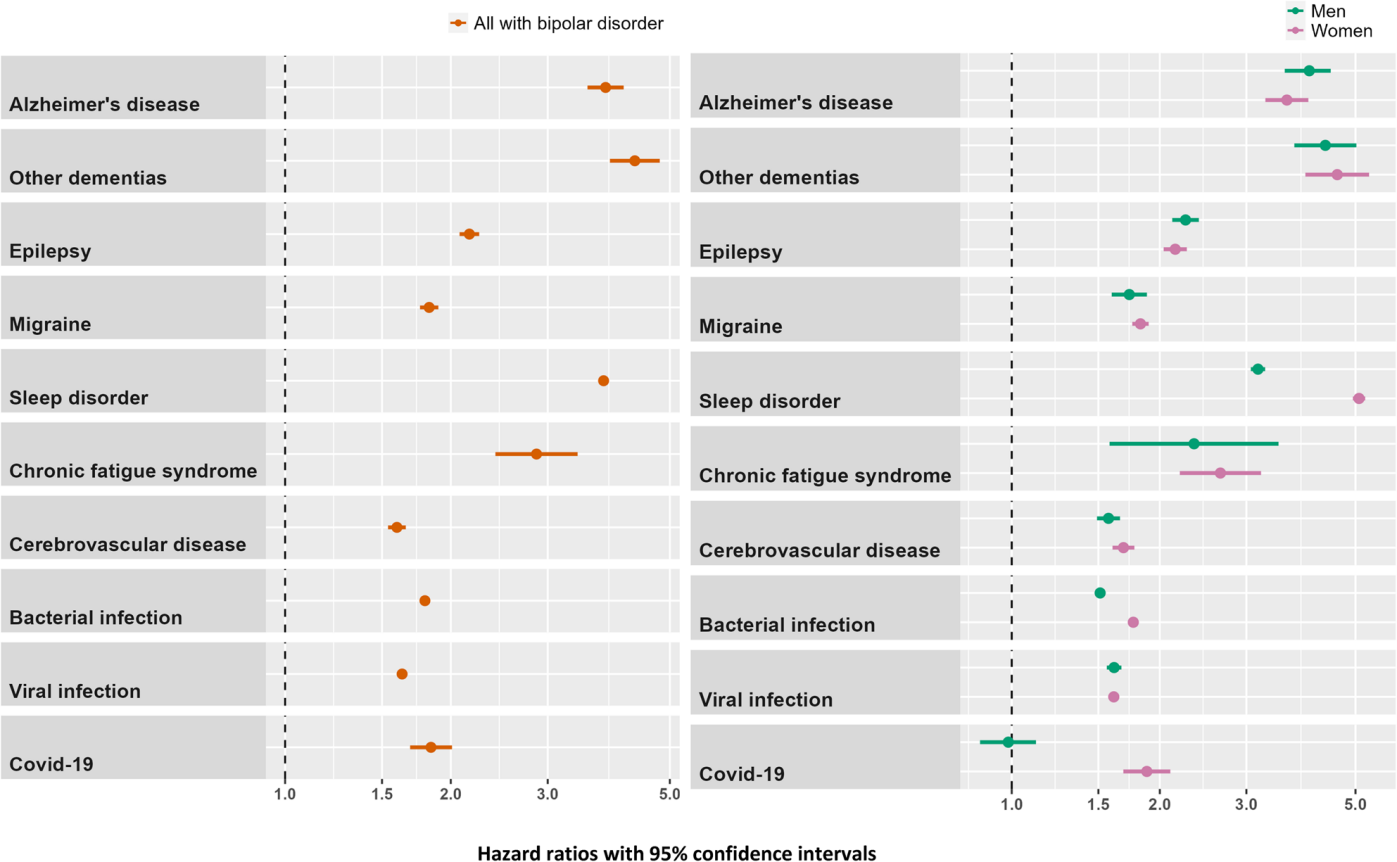
Autoimmune, endocrinological and other conditions in individuals with bipolar disorder (N=61,071) compared with the background population (N=7,912,020), in men with bipolar disorder (N=22,914) and without bipolar disorder (N=4,077,855) and women with (N=38,157) and without bipolar disorder (N=3,834,165)
