# Supplementary material for: Somatic morbidity in bipolar disorders
Source: Int J Bipolar Disord. 2026 May 8;14:19. doi: 10.1186/s40345-026-00427-9 (PMC13156340; doi:10.1186/s40345-026-00427-9)
Supplement: Supplementary file 1 — Supplementary Material 1 [file 40345_2026_427_MOESM1_ESM.pdf]

Supplementary material

Supplementary Table 1. Associations between bipolar disorder and somatic outcomes. Results presented as hazard ratios (HR) with 95% confidence intervals adjusted for parental highest educational level, region and year of birth. HRs compare individuals with bipolar disorder (N=61,071; women N=38,157) to individuals without bipolar disorder diagnosis (N=7,912,020; women N=3,834,165), and also compare those who experienced compulsory care (N=6,748; women N=3,801) to those with no diagnosis.

Outcomes	All with bipolar disorder (N=61,071) HR (95% CI)	Men with bipolar disorder (N=22,914) HR (95% CI)	Women with bipolar disorder (N=38,157) HR (95% CI)	All with bipolar disorder and compulsory care (N=6,748) HR (95% CI)	Men with bipolar disorder and compulsory care (N=2,947) HR (95% CI)	Women with bipolar disorder and compulsory care (N=3,801) HR (95% CI)
**Cardiovascular conditions**						
Any cardiovascular disease	1.45 (1.42–1.48)	1.39 (1.35–1.43)	1.59 (1.54–1.64)	1.65 (1.56–1.74)	1.57 (1.45–1.70)	1.79 (1.66–1.94)
Hyperlipidemia	1.28 (1.23–1.33)	1.21 (1.15–1.28)	1.50 (1.41–1.58)	1.22 (1.09–1.36)	1.24 (1.07–1.45)	1.25 (1.06–1.48)
Hypertensive diseases	1.41 (1.37–1.44)	1.37 (1.33–1.42)	1.49 (1.45–1.54)	1.65 (1.55–1.75)	1.65 (1.52–1.80)	1.64 (1.51–1.79)
Arteriosclerosis	1.30 (1.22–1.38)	1.20 (1.10–1.32)	1.45 (1.34–1.58)	1.12 (0.93–1.34)	1.15 (0.90–1.48)	1.11 (0.85–1.44)
Ischemic heart disease	1.26 (1.21–1.31)	1.20 (1.13–1.27)	1.57 (1.47–1.67)	1.17 (1.04–1.32)	1.13 (0.96–1.32)	1.38 (1.15–1.66)
Arrhythmias	1.21 (1.16–1.25)	1.16 (1.10–1.22)	1.38 (1.31–1.46)	1.55 (1.41–1.70)	1.42 (1.25–1.62)	1.84 (1.62–2.10)
Heart failure	1.63 (1.55–1.71)	1.58 (1.48–1.70)	1.89 (1.76–2.03)	2.12 (1.88–2.40)	1.85 (1.55–2.20)	2.71 (2.29–3.22)
Thromboembolic disease	1.71 (1.64–1.78)	1.64 (1.54–1.75)	1.71 (1.63–1.80)	2.34 (2.12–2.58)	2.22 (1.91–2.58)	2.35 (2.07–2.67)
**Infectious, inflammatory or autoimmune conditions**						
Bacterial infection	1.79 (1.77–1.82)	1.51 (1.48–1.55)	1.77 (1.75–1.79)	1.87 (1.81–1.94)	1.72 (1.63–1.83)	1.79 (1.72–1.87)
Viral infection	1.63 (1.60–1.66)	1.61 (1.56–1.67)	1.61 (1.57–1.65)	1.88 (1.78–1.99)	1.73 (1.59–1.90)	1.86 (1.73–2.00)
COVID-19 infection	1.84 (1.69–2.01)	1.88 (1.69–2.10)	1.70 (1.47–1.96)	2.39 (1.92–2.97)	2.62 (1.90–3.62)	2.20 (1.63–2.96)
Autoimmune disease	1.47 (1.43–1.50)	1.43 (1.37–1.49)	1.38 (1.34–1.42)	1.47 (1.37–1.58)	1.41 (1.25–1.59)	1.39 (1.27–1.51)
Asthma	1.67 (1.63–1.71)	1.41 (1.34–1.48)	1.78 (1.73–1.84)	1.68 (1.55–1.81)	1.22 (1.06–1.40)	1.87 (1.70–2.04)
Type 1 diabetes NPR	1.47 (1.40–1.55)	1.52 (1.41–1.64)	1.51 (1.41–1.62)	1.72 (1.50–1.98)	1.79 (1.48–2.16)	1.67 (1.37–2.04)
Type 1 diabetes NDR	0.97 (0.89–1.05)	1.07 (0.96–1.20)	0.94 (0.84–1.04)	1.05 (0.83–1.33)	1.36 (1.01–1.83)	0.75 (0.50–1.10)
Type 2 diabetes NDR	1.66 (1.61–1.70)	1.60 (1.54–1.66)	1.88 (1.81–1.96)	2.09 (1.94–2.26)	1.68 (1.50–1.89)	2.59 (2.34–2.87)
**Neurological conditions**						
Alzheimer disease	3.82 (3.54–4.12)	4.03 (3.59–4.52)	3.63 (3.28–4.01)	7.22 (6.19–8.46)	7.87 (6.16–10.06)	6.70 (5.44–8.25)
Other dementias	4.32 (3.82–4.79)	4.34 (3.75–5.02)	4.59 (3.95–5.33)	6.93 (5.49–8.74)	7.09 (5.10–9.85)	7.39 (5.31–10.27)
Epilepsy	2.16 (2.08–2.25)	2.26 (2.12–2.40)	2.15 (2.04–2.27)	2.96 (2.67–3.28)	3.01 (2.59–3.50)	3.10 (2.69–3.56)
Migraine	1.83 (1.76–1.90)	1.73 (1.60–1.88)	1.83 (1.76–1.90)	1.56 (1.39–1.76)	1.57 (1.24–1.98)	1.41 (1.23–1.61)
Sleep disorders	3.79 (3.71–3.87)	3.17 (3.06–3.28)	5.08 (4.94–5.23)	4.66 (4.40–4.94)	3.70 (3.39–4.03)	6.34 (5.87–6.85)
Cerebrovascular disease	1.60 (1.54–1.66)	1.57 (1.49–1.66)	1.69 (1.60–1.78)	1.67 (1.51–1.84)	1.80 (1.57–2.07)	1.59 (1.38–1.84)
**Other somatic conditions**						
Polycystic ovary syndrome	N/A	N/A	1.65 (1.55–1.76)	N/A	N/A	1.88 (1.54–2.30)
Obesity	2.55 (2.48–2.61)	2.25 (2.13–2.38)	2.24 (2.18–2.30)	2.52 (2.34–2.71)	2.39 (2.08–2.76)	2.06 (1.89–2.26)
Restless legs syndrome	3.32 (2.98–3.70)	3.00 (2.46–3.66)	3.23 (2.84–3.68)	3.00 (2.16–4.16)	3.34 (1.98–5.64)	2.64 (1.74–4.01)
Irritable bowel syndrome	2.54 (2.44–2.65)	2.10 (1.90–2.32)	2.29 (2.19–2.40)	1.83 (1.58–2.12)	1.54 (1.12–2.11)	1.74 (1.48–2.05)
Fibromyalgia	3.90 (3.69–4.11)	4.72 (3.67–6.07)	3.16 (2.99–3.34)	2.18 (1.78–2.67)	3.10 (1.39–6.92)	1.64 (1.33–2.02)
Chronic fatigue syndrome	2.86 (2.41–3.40)	2.35 (1.58–3.49)	2.63 (2.18–3.19)	1.54 (0.77–3.08)	2.21 (0.71–6.85)	1.23 (0.51–2.95)

Abbreviations: N/A=not applicable; NPR=The National Patient Register; NDR=The National Diabetes Register.

Supplementary Table 2. Time-varying model considering date of first diagnosis as start of exposure to bipolar disorder. Individuals born 1932 or later with bipolar disorder (N=61,071; women N=38,157) and without bipolar disorder (N=7,912,020; women N=3,834,165). Results presented as hazard ratios (HR) with 95% confidence intervals adjusted for highest educational level and year of birth. Not including COVID-19.

**Outcomes**	**All with bipolar disorder (N=61,071) HR (95% CI)** Adjusted for educational level and birth year	**Men with bipolar disorder (N=22,914) HR (95% CI)** Adjusted for educational level and birth year	**Women with bipolar disorder (N=38,157) HR (95% CI)** Adjusted for educational level and birth year
**Cardiovascular conditions**			
Cardiovascular disease	1.47 (1.43–1.51)	1.42 (1.37–1.48)	1.62 (1.56–1.68)
Hyperlipidemia	1.18 (1.13–1.23)	1.12 (1.05–1.19)	1.37 (1.28–1.46)
Hypertensive diseases	1.28 (1.24–1.31)	1.24 (1.19–1.30)	1.35 (1.30–1.41)
Arteriosclerosis	1.27 (1.19–1.37)	1.21 (1.10–1.34)	1.41 (1.27–1.55)
Peripheral artery disease	2.22 (2.14–2.30)	2.00 (1.89–2.11)	2.68 (2.54–2.82)
Ischemic heart disease	1.22 (1.16–1.28)	1.16 (1.08–1.24)	1.52 (1.41–1.64)
Arrhythmias	1.20 (1.15–1.25)	1.15 (1.08–1.22)	1.38 (1.30–1.47)
Heart failure	1.75 (1.66–1.84)	1.64 (1.52–1.77)	2.09 (1.93–2-26)
Thromboembolic disease	1.69 (1.61–1.78)	1.68 (1.56–1.81)	1.68 (1.57–1.79)
**Infectious, inflammatory, or autoimmune conditions**			
Bacterial infection	1.75 (1.72–1.79)	1.57 (1.51–1.62)	1.67 (1.63–1.71)
Viral infection	1.73 (1.67–1.79)	1.65 (1.56–1.75)	1.70 (1.63–1.77)
Autoimmune disease	1.49 (1.44–1.54)	1.45 (1.37–1.55)	1.37 (1.31–1.43)
Asthma	1.63 (1.55–1.70)	1.24 (1.13–1.36)	1.65 (1.57–1.74)
Type 1 diabetes (NPR)	1.60 (1.47–1.74)	1.55 (1.37–1.75)	1.79 (1.59–2.01)
Type 1 diabetes (NDR)	0.71 (0.61–0.82)	0.85 (0.69–1.05)	0.64 (0.52–0.80)
Type 2 diabetes (NDR)	1.78 (1.72–1.83)	1.66 (1.58–1.74)	2.05 (1.96–2.14)
**Neurological conditions**			
Alzheimer disease	3.87 (3.58–4.19)	4.08 (3.61–4.61)	3.67 (3.31–4.08)
Other dementias	4.24 (3.79–4.75)	4.29 (3.67–5.03)	4.47 (3.80–5.26)
Epilepsy	3.14 (2.95–3.34)	3.28 (3.00–3.59)	3.09 (2.84–3.36)
Migraine	1.72 (1.63–1.81)	1.28 (1.12–1.47)	1.52 (1.43–1.61)
Sleep disorders	3.70 (3.60–3.80)	2.98 (2.85–3.11)	5.02 (4.85–5.21)
Cerebrovascular diseases	1.66 (1.59–1.73)	1.62 (1.52–1.72)	1.77 (1.66–1.87)
**Other somatic conditions**			
Polycystic ovary syndrome	N/A	1.50 (1.37–1.65)	N/A
Obesity	2.34 (2.27–2.42)	2.24 (2.09–2.40)	1.90 (1.83–1.98)
Restless legs syndrome	2.80 (2.44–3.22)	2.35 (1.80–3.06)	2.79 (2.37–3.28)
Irritable bowel syndrome	2.06 (1.93–2.19)	1.40 (1.93–1.65)	1.89 (1.77–2.02)
Fibromyalgia	3.20 (3.00–3.41)	4.05 (3.03–5.42)	2.44 (2.28–2.61)
Chronic fatigue syndrome	2.88 (2.31–3.58)	2.53 (1.52–4.21)	2.52 (1.98–3.21)

Abbreviations: N/A=not applicable; NPR=The National Patient Register; NDR=The National Diabetes Register.

Supplementary Table 3. Sensitivity analysis considering only main diagnosis of each outcome. Individuals born 1932 or later with bipolar disorder (N=61,071; women N=38,157) and without bipolar disorder (N=7,912,020; women N=3,834,165). Results presented as hazard ratios (HR) with 95% confidence intervals adjusted for highest educational level and year of birth. Not including COVID-19.

**Outcomes**	**All with bipolar disorder (N=61,071) HR (95% CI)** Adjusted for educational level and birth year	**Men with bipolar disorder (N=22,914) HR (95% CI)** Adjusted for educational level and birth year	**Women with bipolar disorder (N=38,157) HR (95% CI)** Adjusted for educational level and birth year
**Cardiovascular conditions**			
Cardiovascular disease	1.36 (1.33–1.39)	1.31 (1.27–1.36)	1.49 (1.45–1.54)
Hyperlipidemia	1.15 (1.02–1.30)	1.26 (1.07–1.48)	1.13 (0.94–1.34)
Hypertensive diseases	1.18 (1.13–1.24)	1.16 (1.09–1.24)	1.24 (1.17–1.32)
Arteriosclerosis	1.25 (1.17–1.34)	1.19 (1.08–1.31)	1.37 (1.24–1.50)
Ischemic heart disease	1.15 (1.10–1.20)	1.11 (1.04–1.18)	1.42 (1.33–1.53)
Arrhythmias	1.07 (1.03–1.12)	1.02 (0.96–1.08)	1.23 (1.16–1.30)
Heart failure	1.55 (1.45–1.65)	1.57 (1.44–1.70)	1.72 (1.57–1.89)
Thromboembolic disease	1.69 (1.62–1.77)	1.65 (1.54–1.76)	1.69 (1.59–1.78)
**Infectious, inflammatory, or autoimmune conditions**			
Bacterial infection	1.77 (1.75–1.79)	1.49 (1.45–1.52)	1.77 (1.74–1.79)
Viral infection	1.60 (1.57–1.64)	1.59 (1.53–1.65)	1.59 (1.54–1.63)
Autoimmune disease	1.39 (1.35–1.43)	1.36 (1.30–1.43)	1.30 (1.26–1.34)
Asthma	1.50 (1.46–1.55)	1.34 (1.27–1.41)	1.60 (1.55–1.66)
Type 1 diabetes	1.25 (1.17–1.33)	1.28 (1.17–1.40)	1.30 (1.19–1.41)
**Neurological conditions**			
Alzheimer disease	3.12 (2.82–3.44)	3.34 (2.87–3.89)	2.91 (2.55–3.31)
Other dementias	4.11 (3.57–4.73)	4.03 (3.31–4.91)	4.50 (3.68–5.50)
Epilepsy	2.01 (1.92–2.11)	2.11 (1.97–2.26)	2.00 (1.88–2.12)
Migraine	1.96 (1.89–2.04)	1.69 (1.54–1.85)	1.76 (1.68–1.84)
Sleep disorders	3.09 (3.01–3.17)	2.67 (2.56–2.77)	4.31 (4.15–4.47)
Cerebrovascular diseases	1.45 (1.39–1.50)	1.40 (1.33–1.49)	1.55 (1.47–1.63)
**Other somatic conditions**			
Polycystic ovary syndrome	N/A	1.77 (1.65–1.91)	N/A
Obesity	2.90 (2.81–3.00)	2.21 (2.05–2.39)	2.72 (2.63–2.82)
Restless legs syndrome	2.79 (2.40–3.24)	2.53 (1.93–3.33)	2.73 (2.28–3.28)
Irritable bowel syndrome	2.46 (2.35–2.58)	2.06 (1.85–2.30)	2.22 (2.11–2.34)
Fibromyalgia	3.76 (3.49–4.06)	4.35 (3.07–6.15)	2.98 (2.76–3.23)
Chronic fatigue syndrome	2.51 (2.04–3.09)	1.97 (1.22–3.18)	2.35 (1.87–2.96)

Abbreviations: N/A=not applicable; NPR=The National Patient Register; NDR=The National Diabetes Register.

Supplementary Table 4. Definitions of diagnostic codes included under each outcome.

**Outcomes**	**Codes** (according to The International Classification of Diseases (ICD))
Cardiovascular disease	**ICD-8**: 400, 401, 402, 403, 404, 410, 411, 412, 413, 414, 428, 430, 431, 432, 433, 434, 436, 437, 438, 440, 441, 442, 443, 444, 450, 451<br>**ICD 9:** 401, 402, 403, 404, 405, 410, 411, 412, 413, 414, 426B, 427, 428, 430, 431, 432, 433, 434, 436, 437, 438, 440, 441, 442, 443, 444, 451<br>**ICD 10:** I20, I21, I22, I23, I24, I25, I26, I42, I441, I442, I46, I470, I471, I472, I48, I490, I495, I498, I50, I6, I70, I71, I72, I73, I74, I80
Hyperlipidemia	**ICD 8:** 279<br>**ICD 9:** 272<br>**ICD 10:** E78
Hypertensive diseases	**ICD 8:** 400, 401, 402, 403, 404<br>**ICD 9:** 401, 402, 403, 404, 405<br>**ICD 10:** I1
Arteriosclerosis	**ICD 8:** 440, 441, 442, 443, 444<br>**ICD 9:** 440, 441, 441, 442, 443, 444<br>**ICD 10:** I70, I71, I72, I73, I74
Ischemic heart diseases	**ICD 8:** 410, 411, 412, 413, 414<br>**ICD 9:** 410, 411, 412, 413, 414<br>**ICD 10:** I20 I21 I22 I23 I24 I251<br>I252 I255 I256 I258 I259
Arrhythmias	**ICD 8:** -<br>**ICD 9:** 426A, 426B, 427A, 427B,<br>427D, 427E, 427F, 427W<br>**ICD 10:** I441, I442, I46, I470, I471, I472, I48, I490, I495, I498
Heart failure	**ICD 8:** 428<br>**ICD 9:** 428<br>**ICD 10:** I50, I42
Cerebrovascular diseases	**ICD 8:** 43<br>**ICD 9:** 43<br>**ICD 10:** I6, G45
Thromboembolic disease	**ICD 8:** 450, 451<br>**ICD 9:** 415B, 451B<br>**ICD 10:** I26, I80
Bacterial infections	**ICD 8:** -<br>**ICD 9:** 001, 002, 003, 004, 005, 008A, 008B, 008C, 008D, 008E, 008F, 01, 02, 030, 031, 032, 033, 034B, 035, 036, 037, 038, 039, 041, 073, 076, 077A, 078D, 078J, 080, 081, 082, 083, 087, 091, 092, 093, 094, 095, 096, 097, 098, 099A, 099B, 099C, 100, 101, 102, 103, 104, 320, 324, 325, 326, 382, 390, 391, 475, 481, 482, 510, 513, 540, 541, 542, 590, 595, 597A, 599A, 614, 615, 616, 646F, 646G, 68, 711A, 711E, 790H<br>**ICD 10:** A00, A01, A02, A03, A04, A05, A1, A2, A30, A31, A32, A34, A35, A36, A37, A38, A39, A4, A51, A52, A53, A54, A55, A56, A57, A58, A65, A66, A67, A68, A69, A7, B95, B96, G00, G01, G042, G050, G06, G07, G08, G09, H66, H670, I00, I01, J13, J14, J15, J170, J200, J201, J202, J36, J390, J391, J85, J86, K35, K36, K37, L0, M00, M010, M011, M012, M013, N10, N11, N12, N30, N340, N390, N7, O23
Viral infections	**ICD 8:** -<br>**ICD 9:** 466A, 466B, 478B, 478C<br>**ICD 10:** J200, J201, J202, J390, J391
COVID-19	**ICD 10:** G258
Any autoimmune disease	**ICD 8:** 0341, 13607, 24200, 24503, 26910, 28700, 28710, 25810, 390, 391, 392, 340, 35401, 44609, 44630, 44638, 44640, 56300, 56310, 57190, 580, 582, 694, 69610, 69619, 69620, 69621, 69622, 69623, 70400, 71200, 71210, 71239, 71600, 73300, 73400, 73410<br>**ICD 9:** 034B, 136B, 242A, 245C, 258B, 287A, 287D, 340, 357A, 358A, 390, 391, 392, 446A, 446B, 446F, 446G, 555, 580, 582, 556, 571F, 579A, 694A, 694E, 694F, 696, 710A, 710B, 710C, 704A, 710D, 710W, 714A, 725

	**ICD 10:** A389, D686, D690, D693, E050, E063, E310, G04, G131, G35, G610, G700, I00, I01, I02, L100, L120, L13, L40, L63, K900, K50, K51, K743, M06, M300, M301, M32, M311, M315, M339, M34, M350, M352, M353, N00, N01, N03, N05, M303, M317, M351
Asthma	**ICD 8:** 493<br>**ICD 9:** 493<br>**ICD 10:** J45, J46
Type 1 diabetes	**ICD 8:** 250<br>**ICD 9:** 250<br>**ICD 10:** E10
Alzheimer disease	**ICD 8:** 290<br>**ICD 9:** 290A, 290B, 290X, 331A<br>**ICD 10:** F00, F03, G30
Other dementias	**ICD 8:** 2930, 2931<br>**ICD 9:** 290E, 290W, 294B, 331B, 331C, 331X<br>**ICD 10:** F01, F02, F05.1, G31.1, G31.8
Epilepsy	**ICD 8:** 345<br>**ICD 9:** 345<br>**ICD 10:** G40, G41
Migraine	**ICD 8:** 346.09<br>**ICD 9:** 346A, 346B, 346W, 346X<br>**ICD 10:** G43
Polycystic ovary syndrome	**ICD 8:** -<br>**ICD 9:** 256E<br>**ICD 10:** E282
Obesity	**ICD 8:** 277<br>**ICD 9:** 278A, 278B<br>**ICD 10:** E65, E66
Restless legs syndrome	**ICD 8:** -<br>**ICD 9:** -<br>**ICD 10:** G258
Irritable bowel syndrome	**ICD 8:** -<br>**ICD 9:** -<br>**ICD 10:** K58
Sleep disorders	**ICD 8:** 306.40<br>**ICD 9:** 307E, 780F<br>**ICD 10:** G47
Fibromyalgia	**ICD 8:** -<br>**ICD 9:** -<br>**ICD 10:** G258
Chronic fatigue syndrome	**ICD 8:** -<br>**ICD 9:** -<br>**ICD 10:** G933

Supplementary Figure 1. Flow chart of main cohort.

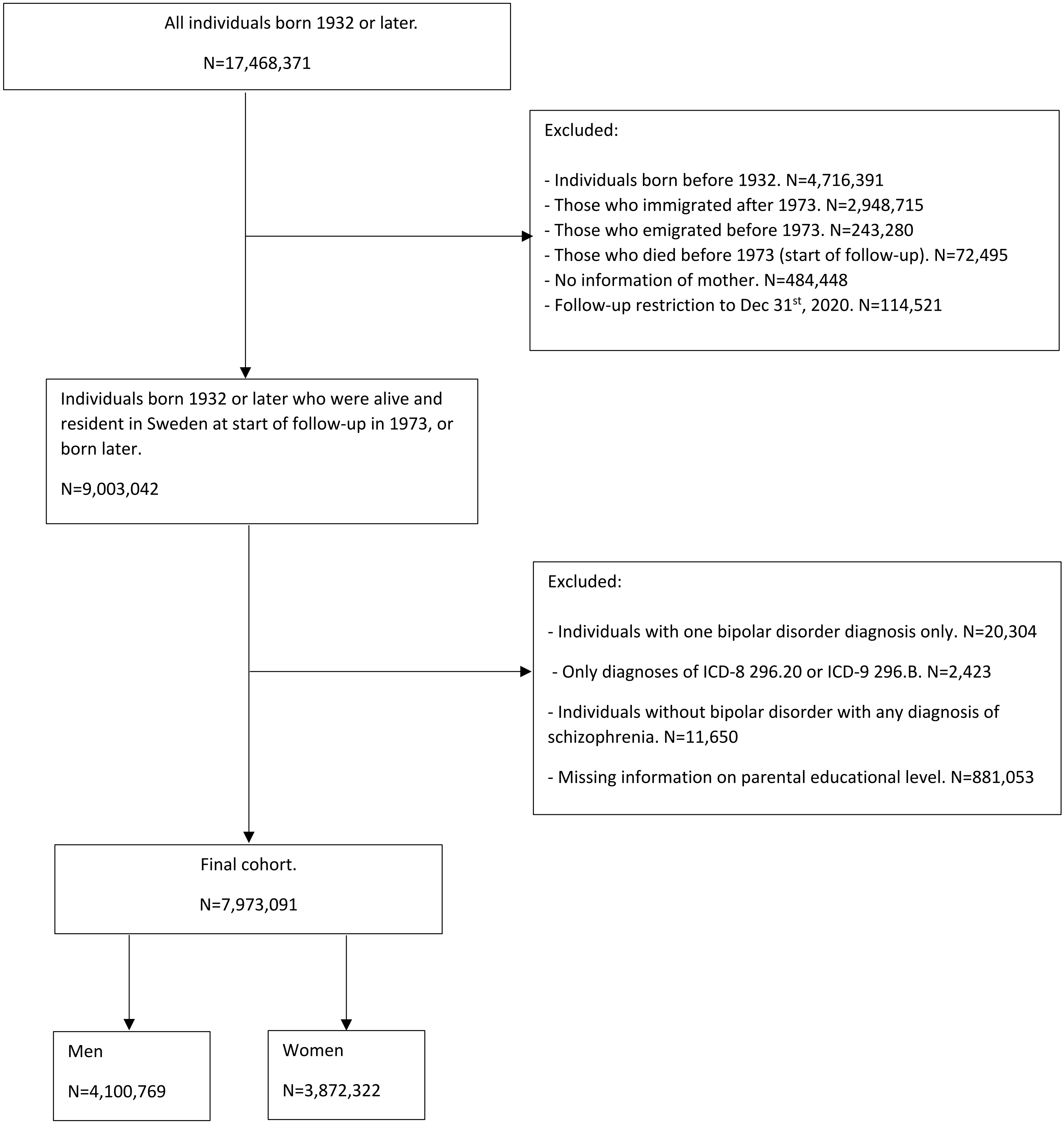

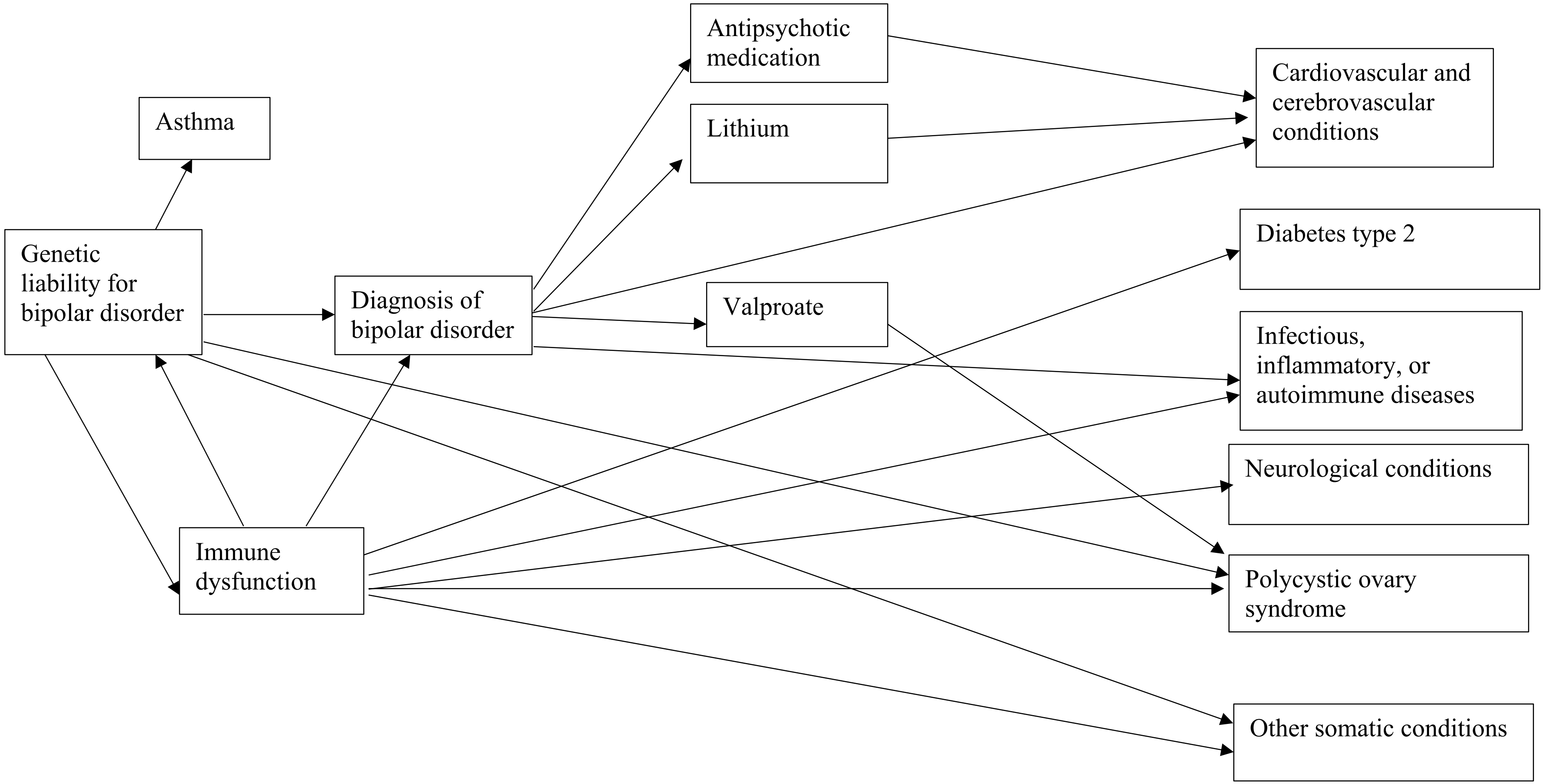
Antipsychotic medication
Lithium
Valproate
Asthma
Genetic liability for bipolar disorder
Diagnosis of bipolar disorder
Immune dysfunction
Cardiovascular and cerebrovascular conditions
Diabetes type 2
Infectious, inflammatory, or autoimmune diseases
Neurological conditions
Polycystic ovary syndrome
Other somatic conditions

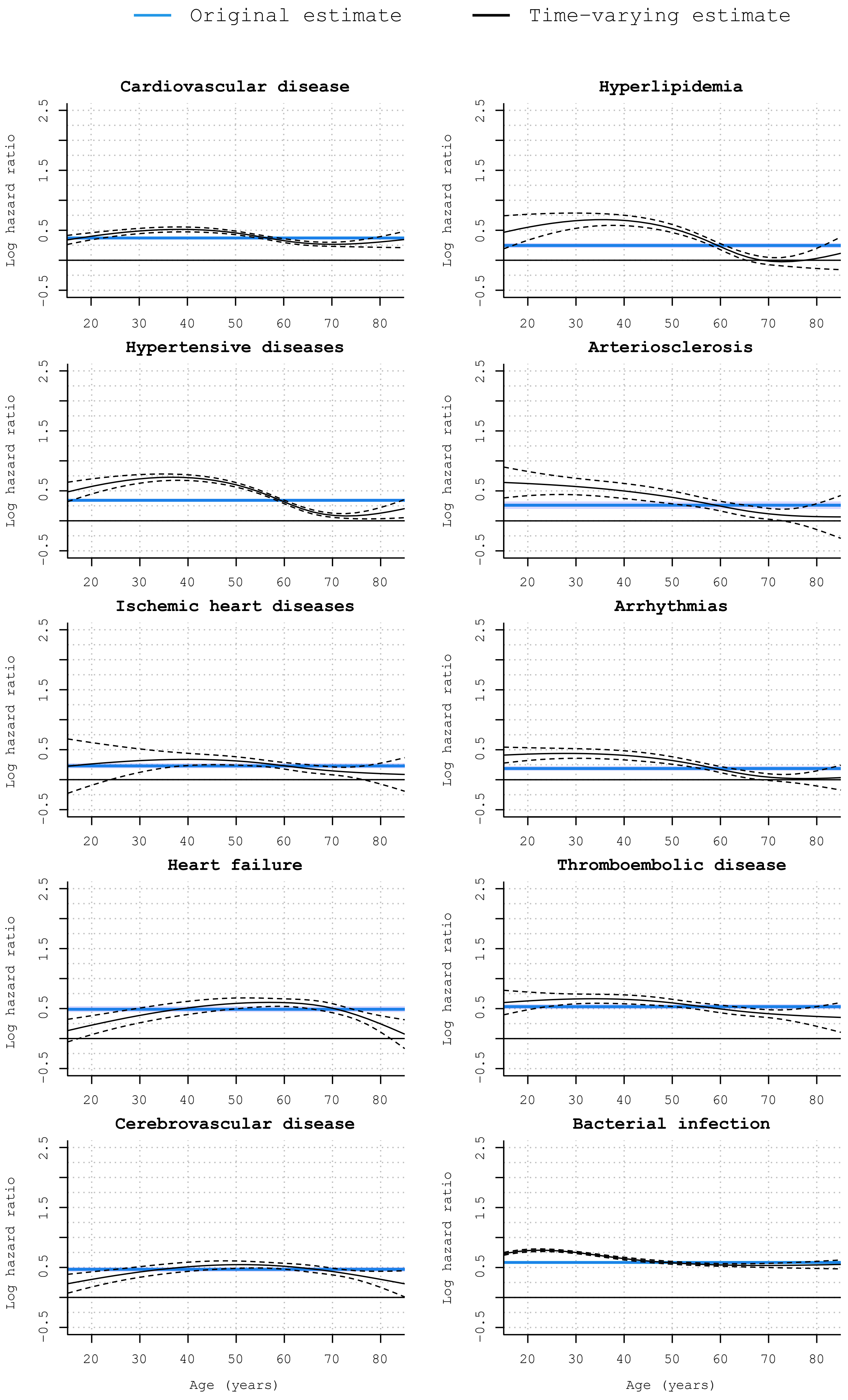

Original estimate
Time-varying estimate
Cardiovascular disease
Hyperlipidemia
Hypertensive diseases
Arteriosclerosis
Ischemic heart diseases
Arrhythmias
Heart failure
Thromboembolic disease
Cerebrovascular disease
Bacterial infection
Log hazard ratio
−0.5
0.5
1.5
2.5
20
30
40
50
60
70
80
Age (years)

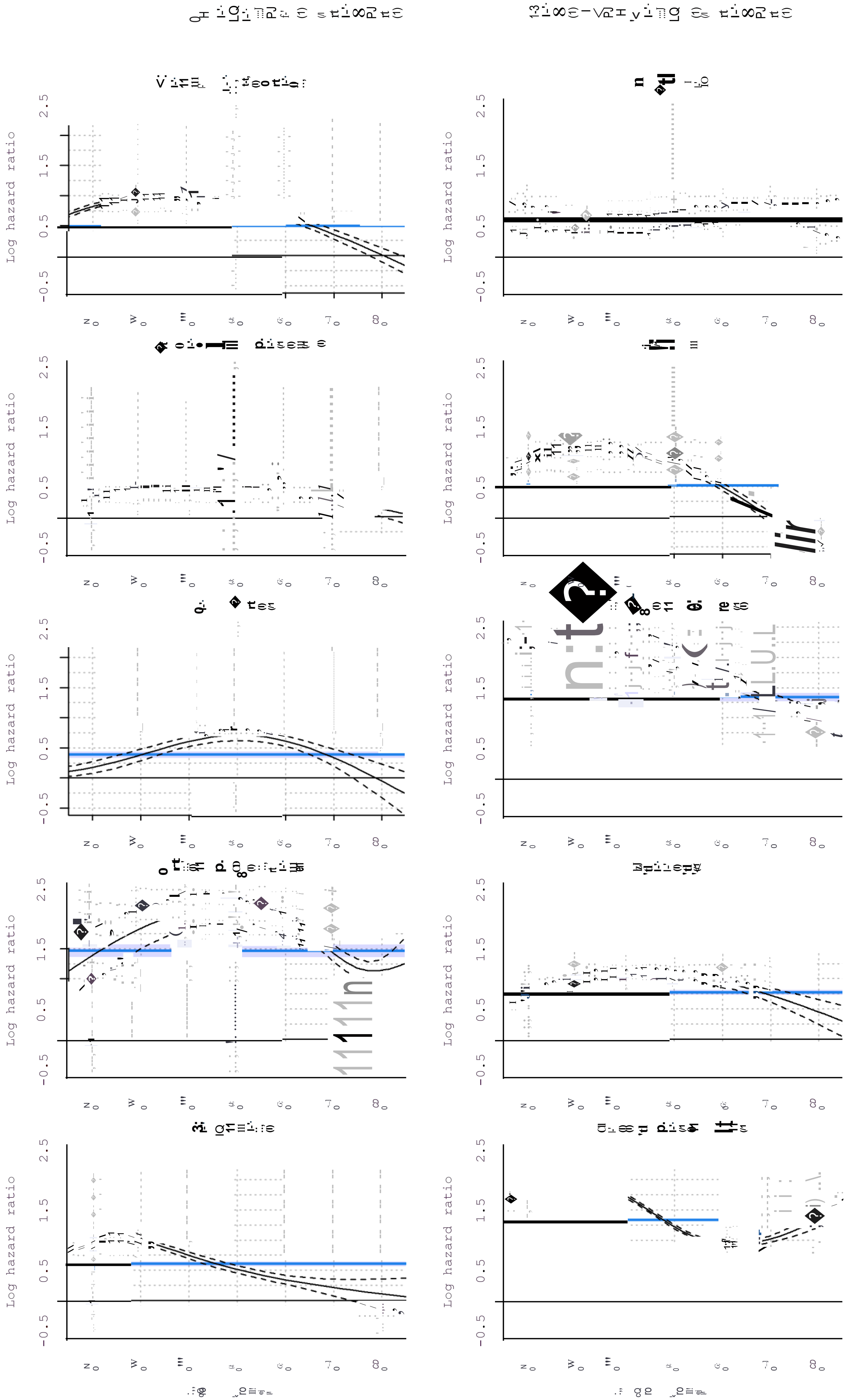
Log hazard ratio
-0.5
0.5
1.5
2.5

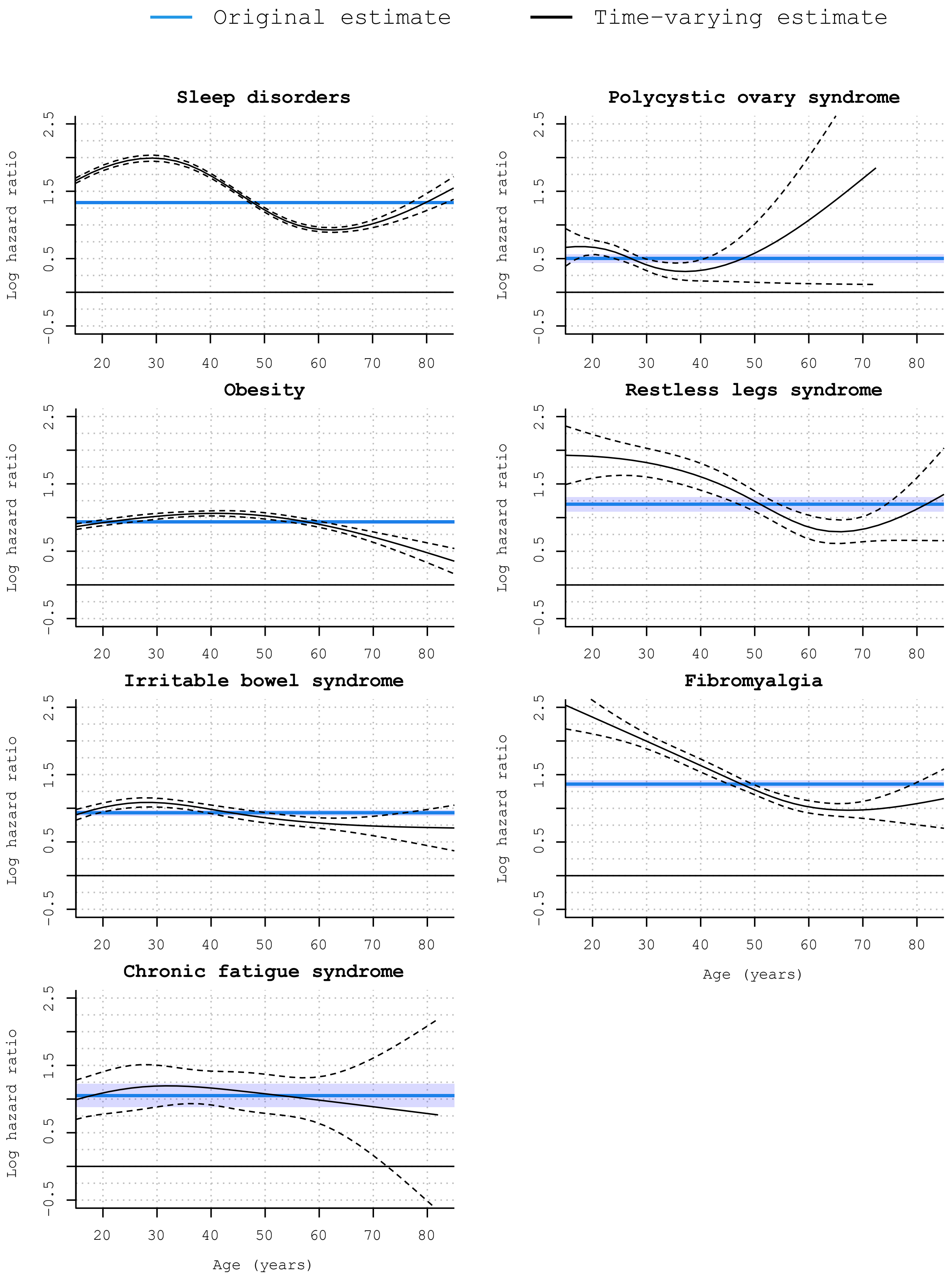

Original estimate
Time-varying estimate
Sleep disorders
Polycystic ovary syndrome
Obesity
Restless legs syndrome
Irritable bowel syndrome
Fibromyalgia
Chronic fatigue syndrome
Log hazard ratio
-0.5
0.5
1.5
2.5
20
30
40
50
60
70
80
Age (years)

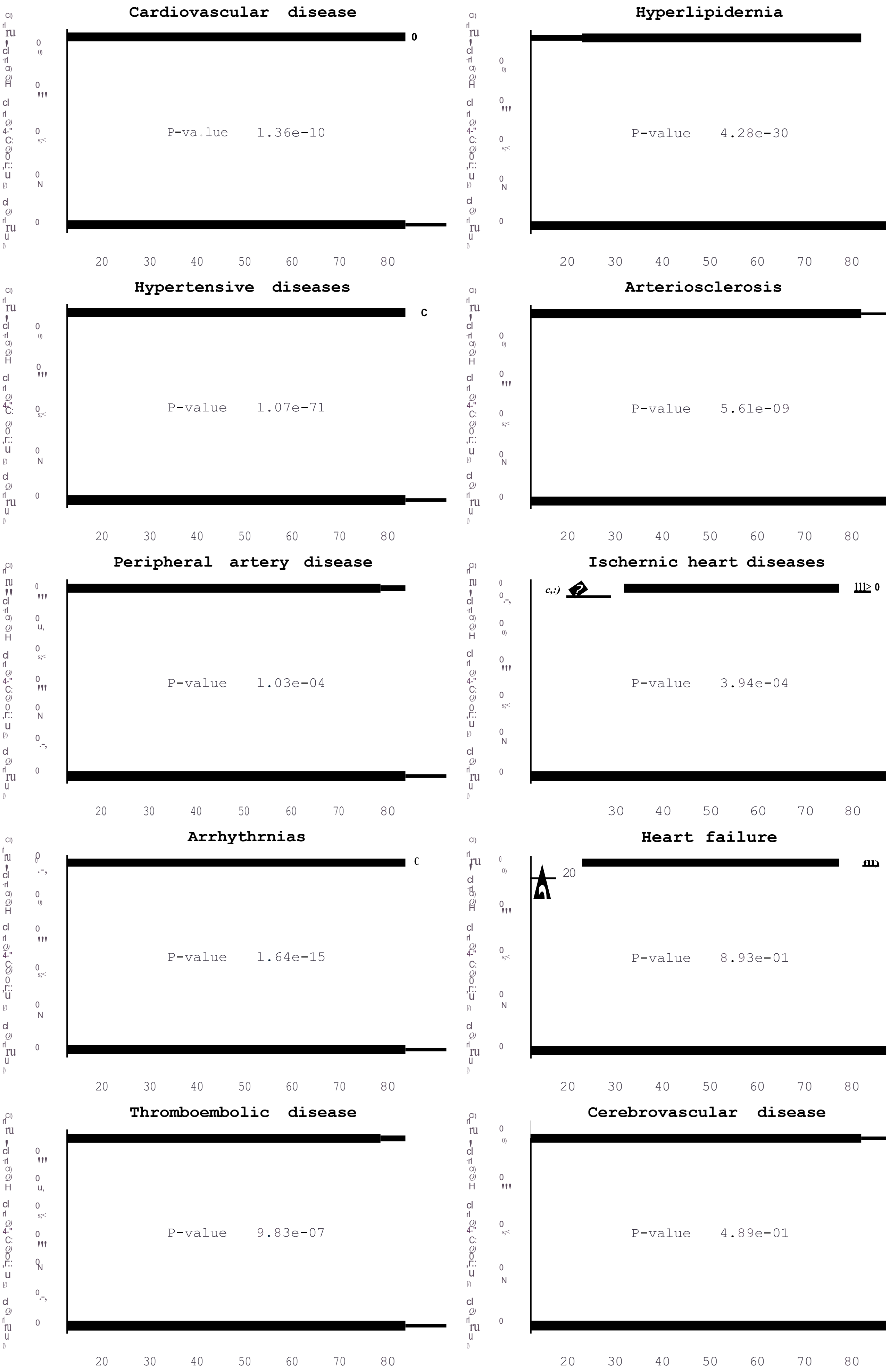

Cardiovascular disease
P-va.lue 1.36e-10
Hyperlipidernia
P-value 4.28e-30
Hypertensive diseases
P-value 1.07e-71
Arteriosclerosis
P-value 5.61e-09
Peripheral artery disease
P-value 1.03e-04
Ischernic heart diseases
P-value 3.94e-04
Arrhythrnias
P-value 1.64e-15
Heart failure
P-value 8.93e-01
Thromboembolic disease
P-value 9.83e-07
Cerebrovascular disease
P-value 4.89e-01
20 30 40 50 60 70 80

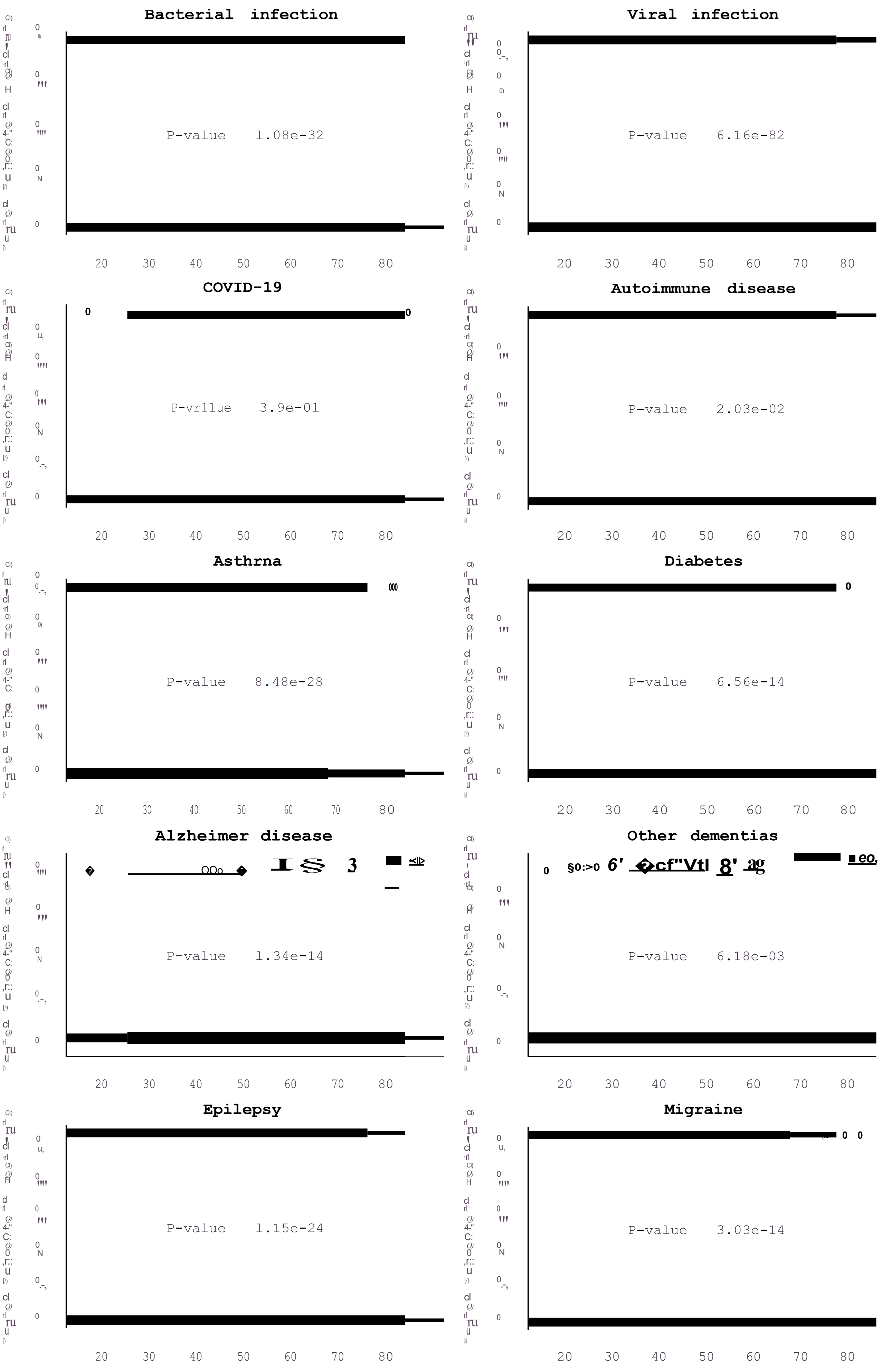

Bacterial infection
P-value 1.08e-32
Viral infection
P-value 6.16e-82
COVID-19
P-vr1lue 3.9e-01
Autoimmune disease
P-value 2.03e-02
Asthrna
P-value 8.48e-28
Diabetes
P-value 6.56e-14
Alzheimer disease
P-value 1.34e-14
Other dementias
P-value 6.18e-03
Epilepsy
P-value 1.15e-24
Migraine
P-value 3.03e-14
20 30 40 50 60 70 80

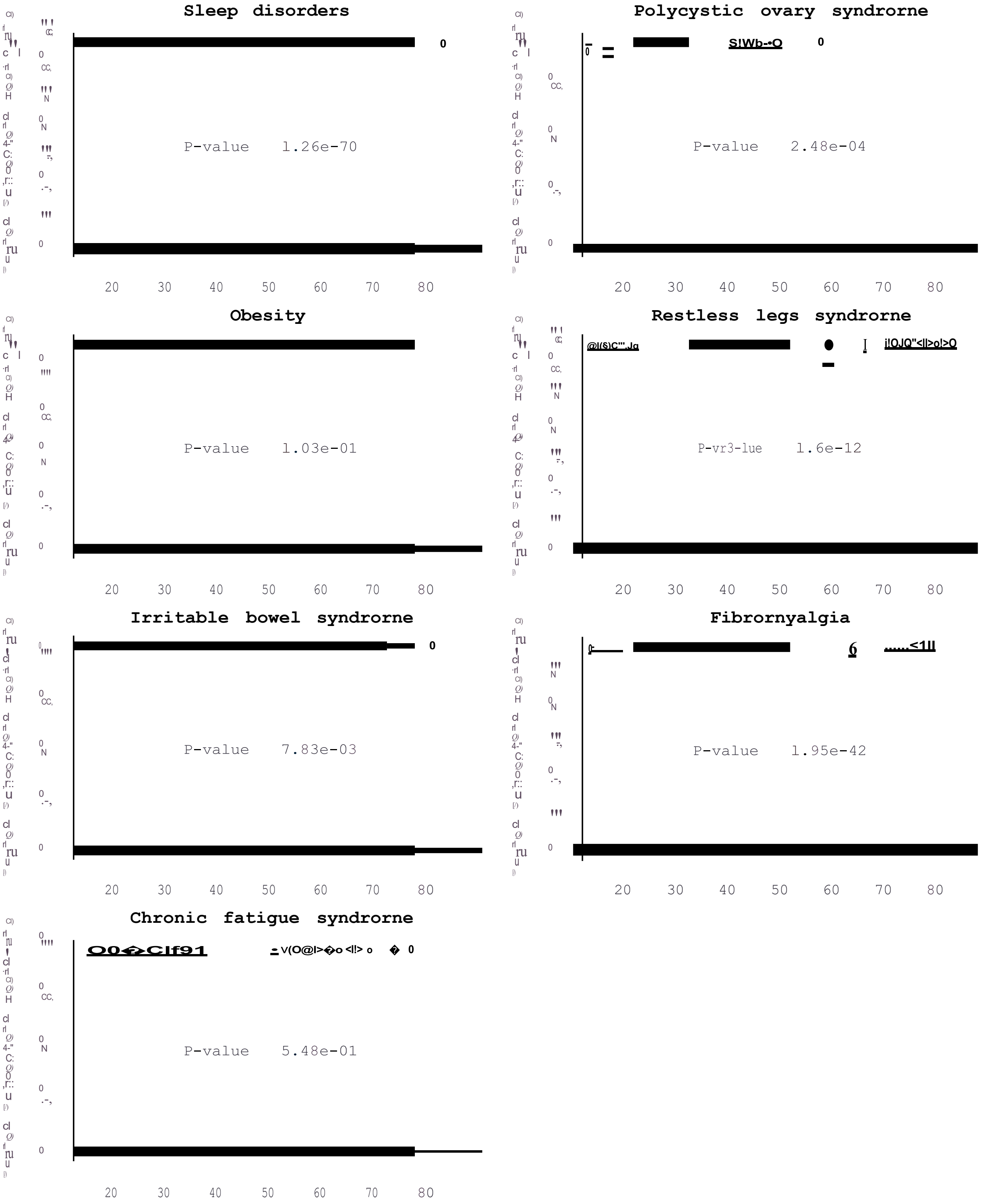
Sleep disorders
P-value 1.26e-70
Polycystic ovary syndrorne
P-value 2.48e-04
Obesity
P-value 1.03e-01
Restless legs syndrorne
P-vr3-lue 1.6e-12
Irritable bowel syndrorne
P-value 7.83e-03
Fibrornyalgia
P-value 1.95e-42
Chronic fatigue syndrorne
P-value 5.48e-01
20 30 40 50 60 70 80
